# EXPRSS: an Illumina based high-throughput expression-profiling method to reveal transcriptional dynamics

**DOI:** 10.1186/1471-2164-15-341

**Published:** 2014-05-06

**Authors:** Ghanasyam Rallapalli, Eric M Kemen, Alexandre Robert-Seilaniantz, Cécile Segonzac, Graham J Etherington, Kee Hoon Sohn, Daniel MacLean, Jonathan D G Jones

**Affiliations:** The Sainsbury Laboratory, Norwich Research Park, NR4 7UH Colney, Norwich, UK; Max-Planck Institute for Plant Breeding Research, Carl-von-Linné-Weg 10, 50829 Köln, Germany; UMR INRA-Agrocampus Ouest-Université de Rennes 1, INRA, UMR1349, IGEPP, BP35327, 35653 Le Rheu Cedex, France; Institute of Agriculture and Environment, Massey University, Palmerston North, 4442 New Zealand

**Keywords:** Next generation sequencing, Tag-seq, High throughput expression profiling, RNA-seq, EXPRSS

## Abstract

**Background:**

Next Generation Sequencing technologies have facilitated differential gene expression analysis through RNA-seq and Tag-seq methods. RNA-seq has biases associated with transcript lengths, lacks uniform coverage of regions in mRNA and requires 10–20 times more reads than a typical Tag-seq. Most existing Tag-seq methods either have biases or not high throughput due to use of restriction enzymes or enzymatic manipulation of 5’ ends of mRNA or use of RNA ligations.

**Results:**

We have developed EXpression Profiling through Randomly Sheared cDNA tag Sequencing (EXPRSS) that employs acoustic waves to randomly shear cDNA and generate sequence tags at a relatively defined position (~150-200 bp) from the 3′ end of each mRNA. Implementation of the method was verified through comparative analysis of expression data generated from EXPRSS, *Nla*III-DGE and Affymetrix microarray and through qPCR quantification of selected genes. EXPRSS is a strand specific and restriction enzyme independent tag sequencing method that does not require cDNA length-based data transformations. EXPRSS is highly reproducible, is high-throughput and it also reveals alternative polyadenylation and polyadenylated antisense transcripts. It is cost-effective using barcoded multiplexing, avoids the biases of existing SAGE and derivative methods and can reveal polyadenylation position from paired-end sequencing.

**Conclusions:**

EXPRSS Tag-seq provides sensitive and reliable gene expression data and enables high-throughput expression profiling with relatively simple downstream analysis.

**Electronic supplementary material:**

The online version of this article (doi:10.1186/1471-2164-15-341) contains supplementary material, which is available to authorized users.

## Background

Gene expression profiling is widely used to investigate the dynamics of cellular responses through quantification of changes in transcript abundances. Microarray methods have been widely used [1] to monitor global gene expression changes during investigations of transcriptional regulatory mechanisms and have been the gold standard in expression profiling studies for most of the last 15 years. Alternate methods, such as sequencing of expressed sequence tags (ESTs) [2] and differential display PCR [3] were restricted by cost and design difficulties. With the advent of Serial Analysis of Gene Expression (SAGE) [4], sequencing-based expression profiling emerged as a cost-effective alternative to microarrays. Despite various improvements like LongSAGE [5] and SuperSAGE [6], issues such as lower data acquisition rates, laborious protocols, and the requirement for specialized equipment have restricted rapid adoption of sequencing-based methods. Recent improved sequencing techniques [7–9] have led to a remarkable increase in data acquisition rates, and have enabled simplified protocols. With wider accessibility of these sequencing systems, there has been an increase in the use of sequencing-based expression profiling methods; these have several advantages, such as the opportunity to detect all expressed genes and to determine their absolute abundances without hybridization biases. Additionally, higher depth of sequencing enables superior dynamic range of detection, highly sensitive differential expression assays, and also an opportunity to minimize costs through multiplexed sequencing.

Recent advances in Next Generation Sequencing (NGS) technologies have been discussed in detail in several reviews [9–11]. NGS technology has facilitated genome re-sequencing [12, 13], de-novo genome assembly [14, 15], transcriptome and non-coding RNA sequencing, transcriptome assembly [16–19], as well as sequencing of genome wide protein binding or methylation sites (ChIP-seq and Methyl-seq) [20–22]. NGS transcriptome sequencing methods are broadly referred to as RNA-seq methods and have also been used for differential gene expression analysis [18]. RNA-seq is a powerful method for whole transcriptome annotation, identification of novel splice junctions and rare transcription events. Several other methods were developed based on the principles of SAGE (LongSAGE, superSAGE and 5′ SAGE) methods that sequence a small fragment of cDNA from a defined position for expression profiling [23–25]. These methods can be broadly referred as Tag-seq methods as they are aimed at sequencing a small and relatively defined region of mRNA generally referred as ‘tags’. RNA-seq can be used to measure differential expression, but the need for 10–20 times more reads than a typical Tag-seq, biases associated with transcript lengths [26], and the absence of uniform coverage of regions in mRNA [27] make inferences about relative gene expression levels difficult. With Tag-seq methods one cDNA tag is sequenced from each expressed gene, thereby making relative expression analysis straightforward by simply counting tags for each gene. Increased depth of sequencing allows investigators to make these methods cost-effective through barcoded-multiplexed sequencing.

Most existing Tag-seq methods such as *Nla*III-DGE (/*Dpn*II-DGE) or Super SAGE or 5′ SAGE methods use restriction enzymes and adapter ligation for cDNA tags. For SAGE to work, each transcript requires the presence of a restriction enzyme recognition site (referred as anchoring enzyme such as *Nla*III or *Dpn*II to provide an anchor site for tag generation). Therefore, no expression information can be obtained for a gene without the anchoring enzyme site. In Arabidopsis, 3 and 5 % of genes lack *Dpn*II and *Nla*III sites respectively. The SAGE methods also involve digestion using a tagging enzyme (MmeI or EcoP15I), which produces a short cDNA tag that is sequenced later. Each of these tagging enzymes has a complex restriction nuclease activity and the mechanism of restriction digestion of these enzymes is not entirely understood. An additional shortcoming of SAGE is that the tagging enzyme sites often occur within the genes and lead to longer tag sequences, resulting in loss of such tags during size selection. For example, in Arabidopsis 14383 genes (out of 33602) have at least one recognition site (TCCRAC) for MmeI in the sense strand. MmeI, a type IIS restriction enzyme, has both restriction and methylating activity and inhibits restriction digestion at high enzyme concentration due to methylation of the recognition site [28]. Similarly EcoP15I, a type III restriction enzyme with restriction and methylation activity, needs two restriction recognition sites in head-to-head orientation to effectively digest the DNA, and the distance between two recognition sites can influence digestion efficiency [29–31]. Methods based on analysis of 5′ ends of cDNAs either involve low throughput methods eg using methyl cap [32] or have amplification biases that are less reliable in strand specificity (methods using SMART cDNA amplification) [33, 34]. There is thus a need for a Tag-seq method that produces reliable expression data with no or minimal biases and is amenable for analysing hundreds of samples.

Others have attempted to generate restriction digestion independent Tag-seq using nebulisation [35]. However, nebulisation for cDNA shearing of many samples is not practical. We used adaptive focussed acoustics (AFA), a technology from Covaris (http://www.covarisinc.com/) that uses high frequency ultrasonic sound waves, to shear DNA to a desired size. AFA-sheared DNA or cDNA has been successfully used for whole genome and whole transcriptome sequencing [36, 37]. We have developed a protocol named EXpression Profiling through Randomly Sheared cDNA tag Sequencing (EXPRSS) that employs AFA random shearing of double-stranded cDNA for high throughput expression profiling. Implementation of EXPRSS tag generation protocol is not limited to AFA sheared cDNA but can also be used for sonicated cDNA derived from other technologies such as SonicMan (http://www.matrical.com/) or Bioruptor (http://www.diagenode.com/).

## Results

### EXPRSS Tag-seq protocol and experimental setup

**Figure 1:**
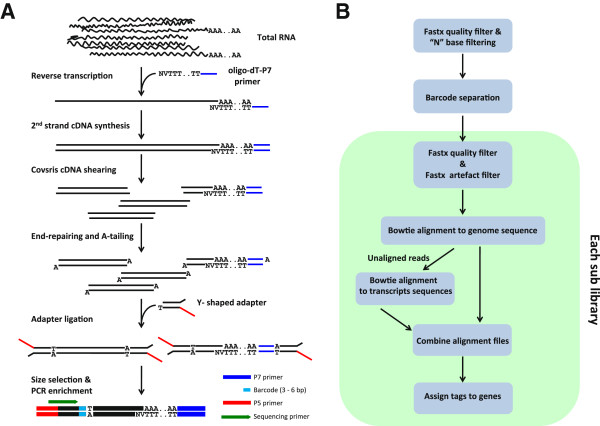
**Schematic diagram representing EXPRSS Tag-seq. (A)** Library preparation **(B)** Data analysis pipeline.

The EXPRSS Tag-seq method involves the following steps (Figure 1). First strand cDNA is synthesized through reverse transcription of poly(A) RNA from total RNA using a custom oligo-dT primer. The oligo-dT primer, at the 5′ end, has Illumina single end flow cell primer sequence referred as P7 [13] and the oligo-dT sequence was terminated with 12 two nucleotide combinations comprising V (AGC) & N (ATGC) to initiate reverse transcription at a poly (A) junction. Second strand cDNA synthesis was carried out according to Okayama and Berg [38]. Purified cDNA was sheared using Covaris AFA to a target size of ~200 bp and sheared cDNA was end-repaired and dA-tailed. A Y-shaped adapter with a dT overhang was ligated to dA-tailed sheared cDNA fragments. The Y-shaped adapters were designed as described previously by Prashar and Weissman [39] so that the primer binding sites for amplification on the adapter end will only be synthesized after priming from the P7 sequence on oligo-dT end. Therefore, the Y-shaped adapters ensure selective amplification of the 3′-most fragment of each cDNA, so that only one tag per transcript is sequenced at a defined average distance from the 3′ end, specified by the choice of shearing parameters. This adapter design also prevents adapter dimer amplification and also provides the opportunity to directly sequence on Illumina without prior amplification. The adapter also contains 3 to 6 base barcodes at the ligation junction for multiplexed sequencing of libraries. Barcodes of different lengths were introduced to avoid predominance of T at any particular base position, resulting from T/A ligation. Alternative implementation of “bareback” from the Babraham Institute [40] would have enabled us to use a simpler barcode design. However, it is not possible to store sequence data images with current data analysis pipeline of GAII and no such option exists for Hiseq. Hence, the approach we adopted is an excellent method for multiplexed sequencing with reliable base calling on the Illumina platform. Alternatively, an unsupported version of the base calling pipeline is available with a delayed base calibration option, which was found to give mean quality of reads less than those from real time analysis [40]. The barcodes were designed so that with an equimolar mixture, there would be near equal distribution of all four bases at each position in the first 5–6 bases. Therefore, it is not necessary to run a PhiX lane for base calibration of cDNA tag sequencing especially for samples with GC content < 40 %, such as the Arabidopsis transcriptome.

To test the EXPRSS method, an experiment was conducted in which leaf discs from 5 week old plants of Arabidopsis ecotype Col-0, were treated either with water or flg22 (22 amino acid peptide from bacterial flagellin) for 60 minutes [41]. Four biological replicates were collected and 5 μg of total RNA was used from each sample for library preparation. For comparative purposes, 5 μg of each RNA sample was also subjected to *Nla*III-DGE library preparation protocol [42, 43]. However three modifications were made to the *Nla*III-DGE protocol (see Additional file 1, Figure S1A & B); (1) barcodes were introduced in the Adapter2 for multiplexed sequencing and (2) methylation of adenosine was used in the *Nla*III recognition site (CATG) to favour Adapter1 ligation to cDNA over cDNA and cDNA ligations [44] and (3) adapter1 was biotinylated to avoid two rounds of size selection to remove Adapter2 self-ligated products, which would get preferentially amplified creating PCR biases in the library [45]. A detailed protocol is provided in Additional file 2. Eight libraries (4 each of control and treatment) prepared for each method, were mixed in equimolar ratios and sequenced using Illumina Genome AnalyzerII (GAII).

### EXPRSS sequence tags can be reliably assigned to transcripts

EXPRSS library preparation uses total RNA and unlike *Nla*III-DGE no prior selection for poly(A) RNA was performed. Most of the total RNA is ribosomal RNA (rRNA), and the number of reads mapped to Arabidopsis rRNA sequences [46, 47] was examined to rule out non-specific reads from rRNA. Reads mapping to rRNA from the eight sequenced EXPRSS libraries accounted to 0.64 %. A similar analysis on *Nla*III-DGE reads indicate about ~0.13 % of reads map to rRNA. Most of the rRNA reads in EXPRSS are from transcripts downstream of the 5.8S rRNA-encoding loci (AT2G01020 and AT3G41979) with cDNA evidence in TAIR database (see Additional file 1, Figure S2). Although *Nla*III-DGE has less reads from rRNA, there were no reads from the transcripts downstream of 5.8 s rRNA-encoding loci, as they lack *Nla*III restriction sites, exemplifying one of the caveats for restriction enzyme based methods.

**Figure 2:**
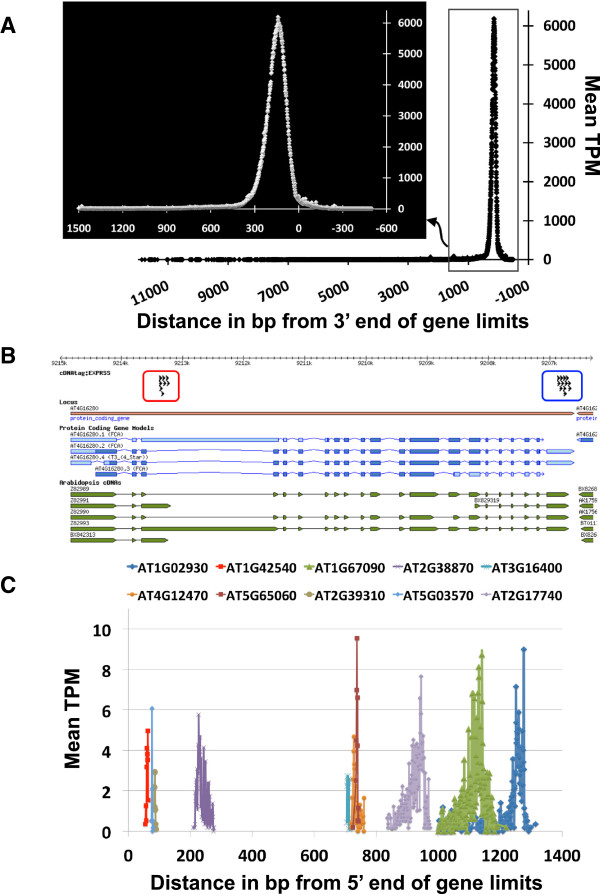
**Characteristics of EXPRSS tag-sequences. (A)** Uniquely aligned tags to the sense strand of cDNA and genome sequences from all Arabidopsis genes are used to plot tag alignment position as a distance from 3′ end of annotated genes against the frequency of reads mapped. Rectangle selection is shown as inset picture. **(B)** Example of alternative polyadenylation. A distinct cluster of tags from both full length (blue circle) and short (red circle) sense transcripts of *FCA* (AT4G16280), which are about 6.5 kb apart. Evidence from cDNA sequences (green arrows) in the TAIR database corroborates short transcripts. Reads from such alternative polyadenylation transcripts result in a long tail in tag alignment frequency distribution presented in A. **(C)** Frequency distribution of antisense tags aligning to 10 selected transcripts plotted against mapping position as a distance from 5′ end of annotated genes. Each individual abundantly expressing an antisense transcript has a distinct peak of tag alignment suggesting a defined polyadenylation site for antisense transcripts identified through EXPRSS method.

Individual libraries from multiplexed samples were aligned to TAIR10 genome sequences [48] using Bowtie short read alignment software [49]. Three main groups of tag sequences were observed from the uniquely mapped reads of EXPRSS data sets (Figure 2). Type A tags (the majority) align within ~300 bp from the 3′ end of existing gene models. Type B tags align upstream of 300 bp from the 3′ end, revealing genes with early polyadenylation sites. Type C tags align to antisense strands of existing gene models. In the following three sub-sections, we explain various sequence tag groups detected and their assignment to gene expression analysis.

i EXPRSS Tag-seq generates cDNA tags at a controlled distance from polyadenylation site

The EXPRSS method is mainly intended to produce a tag sequence from the 3′ end of each cDNA. As the cDNA is randomly sheared to ~200 bp, which includes 48 bp of oligo-dT_P7 primer, the resulting fragments should contain ~150 bp of transcript sequence. If shearing of cDNA is random, most sequenced reads should align within ~300 bp (150 ± 150 bp) from the 3′ end of cDNA. This distribution however, depends on the precision of polyadenylation machinery and the dexterity in fragment size selection. Furthermore, shearing of cDNA overcomes the bias associated with longer genes since to make a tag from cDNA for a gene, only the last 300–400 bp need to be reverse-transcribed. Also, each gene produces only one tag per transcript thus, avoiding the need for length-based data transformations usually employed in quantitative RNA-seq protocols. The efficiency of random shearing was tested by plotting mapping frequency against the alignment distance from the 3′ end of cDNA. Uniquely matched tags to the sense strand of genes were included in this analysis and the distance for the longest splice variant was taken into consideration for tags that match to multiple splice variants of a gene. Figure 2A shows the plot of pooled frequency of tags aligned against the distance from 3′ ends in bp from the start position of the tag. As expected for random shearing, ~90 % of the tags aligned within the ~300 bp distance from 3′ end with a clear peak at ~150 bp. Tags derived from transcript variants, with either premature or alternative polyadenylation, contribute to the long tail of distribution reaching the 5′ end (Figure 2A). Tags aligning to distinct long and short transcripts of *FCA* (cDNA clones are ~6.5 kb apart – Figure 2B) support this interpretation. Identification of alternative polyadenylation sites in EXPRSS data sets is only reliable when these sites are >200 bp apart so that tag peaks can be identified and distinguished. Affymetrix microarray probes for each Arabidopsis gene were designed to span up to ~600 bases upstream of the stop codon [50], which thus also pools expression information from alternative transcripts of a gene as one. Therefore, for expression analysis purposes, both Type A and Type B tags that align to a particular gene and its splice variants are combined. All reads aligning to different splice variants of a gene are pooled.

ii Antisense transcription

The tag sequences resulting from the EXPRSS method are directional. Thus, type C tags aligned to the alternate strand are either due to antisense transcription or novel transcripts on the antisense strand of annotated transcripts. Antisense transcription revealed by SAGE or RNA-seq analysis has been widely reported from plants and animals [51–57]. In the present study, ~ 17% of the transcripts that provide tags (mean expression of ≥1TPM [tags per million] from at least 4 replicates) were found to be derived from the antisense strand of genes. Antisense transcription observed in this study is not due to second strand cDNA synthesis by reverse transcriptase, which was shown to compromise strand specificity of microarray based techniques [58]. Such artefacts would not influence EXPRSS data sets, since the strand synthesized from mRNA using oligo-dT primer acts as a template for sequencing, thus providing the same strand information as that of the mRNA.

One hypothesis for the high amounts of antisense transcription observed is gene looping during transcription [59] which might lead to antisense transcription by jumping of the transcriptional machinery to the other strand. In this scenario, genes with high levels of sense transcription should have a higher chance of antisense transcription. If that were the case, genes with differential expression in both sense and antisense transcripts (263 upregulated and 34 downregulated genes - Table 1), would be expected to show high correlation between sense and antisense counterparts and relatively high expression level of the sense transcripts (upregulated genes in treated sample and downregulated genes in control). However, this was not the case, as the pairwise correlation of mean expression represented as TPM for the 297 genes between sense and antisense transcripts showed that apart from a few genes on the diagonal and genes with high expression, the distribution was either side of the diagonal (see Additional file 1, Figure S3A). Additionally the distribution of mean expression for the 297 genes shows that about 44% of sense transcripts have less than 250 TPM, while only 6% of the genes have more than 2000 TPM (see Additional file 1, Figure S3B). It has also been suggested that antisense transcription might arise due to convergently overlapping gene pairs (COPs) [51]. Further analysis identified that only 7 (out of 379 antisense genes differentially expressed in the present study) are part of 2147 genes identified as COPs in Arabidopsis by Jen et al. [60]. The majority of genes with antisense transcription had a clear peak of tags at a defined position suggesting a relatively defined polyadenylation position for these transcripts (Figure 2C), supporting a possible regulatory role for these antisense transcripts. These data rule out that antisense transcription is an artefact. Since the current study is focussed on establishing a method for high throughput expression profiling, this antisense transcription was not further investigated.

iii Non canonical transcription and tag to gene assignment

**Table 1:** **Differential expression observed for sense and/or anti-sense transcripts of genes using EXPRSS**

Sense transcript	Antisense transcript	Number of genes
Up regulated	Up regulated	263
Down regulated	Down regulated	34
Up regulated	ND	1175
Down regulated	ND	1032
ND	Up regulated	44
ND	Down regulated	38

ND - No differential expression.

Two other types of tags obtained from this study are sequences from novel polyadenylated longer transcripts and sequences from regions of genome without predicted gene models (novel transcripts). Tags not assigned to any genes from the Arabidopsis genome (TAIR10) alignment were further analysed to identify regions with novel transcription and alternative polyadenylation. A transcript downstream of AT1G02360 shows flg22-dependent downregulation (see Additional file 1, Figure S4A), while a chloroplast-derived transcript upstream of ATCG00270 (see Additional file 1, Figure S4B) shows similar and high expression in all replicates of control and treatment samples. Further instances of novel transcription were identified when all the un-assigned reads from eight libraries were pooled (see Additional file 1, Figure S4C & D), highlighting the need for increased depth of sequencing to investigate such phenomena.

To assign tags to genes, reads were initially mapped to Arabidopsis genome sequences [48] and unaligned reads were mapped to transcript sequences [61]. Details of tag to gene association and alignment parameters are explained in the methods section. We have created modules of our analysis pipeline and implemented them in Galaxy, an open web-based research platform [62] and modules are provided in Additional file 3. EXPRSS Tag-seq can generate long (36 bp to 150 bp) reads. With EXPRSS reads, we found that ~94% of reads aligned uniquely, ~5% mapped to multiple positions and 1% were unmapped. The longer cDNA tags prompted us to test the number of genes each multiply-matched read aligned to. From the plot of cumulative frequency of multiple matched reads against the number of genes, it was striking to observe that ~77% of multiple matched reads aligned only to two genes (see Additional file 1, Figure S5). With Arabidopsis having duplicated at least 2–3 times [63], many genes show paralogous gene families. The EXPRSS data either can reveal differential expression patterns between paralogs or sum expression levels of indistinguishable paralogs. Where tags could be assigned to up to 10 multiple paralogs, matching reads were split equally between them.

### Multiplexing with reproducible tag sequencing

Since our experiments commenced, reads per lane on the Illumina GAII increased from 12 million to 35–40 million reads. This enables ~16 fold multiplexing for 2 million reads per library. We employed barcodes of varying length in EXPRSS, with 8 fold multiplexing for experiments presented in this manuscript and 16 fold multiplexing at present in our lab. For EXPRSS ~12.4 million reads passing the Illumina quality filter were obtained of which ~2.1% reads with “N” and below quality threshold were discarded (see methods section for more details). For *Nla*III-DGE ~13 million reads passing the Illumina quality filter were obtained of which ~10% were discarded as reads with “N”, below quality threshold, adapter only and shorter than 14 bp tag sequence. Sequenced libraries were computationally assigned to sub-libraries based on perfect match to barcode for EXPRSS data and up to 1 mismatch allowed for *Nla*III-DGE data. For EXPRSS, each sub-library received ~12% reads, while 2.1% have no distinct barcode. For *Nla*III-DGE, each library received ~11.1% reads, and 1.4% reads with no distinct barcode were discarded. Library distributions are presented in Table 2. Artefact filtering using FASTX-toolkit [64] resulted in loss of 7.7% and 4% of reads in each sub-library for EXPRSS and *Nla*III-DGE respectively. Most of these reads were found to be due to poly(A) sequences, initially assumed to be resulting from the poly(A) tail. On further inspection these reads in EXPRSS were found to result from ligation of excess oligo-dT primer to the Adapters. However, this factor introduced no technical bias into EXPRSS libraries as evident from higher reproducibility of replicates presented later. Since these fragments occur due to T/A ligations, we have designed an approach to reduce oligo-dT primers ending with Adenosine. Since our oligo-dT primer is a mixture of 12 primers ending with VN (V-A, C & G and N-A, T, G & C) we have used an equimolar mixture of 9 primers ending with V*B (B-T, G & C and * - is a phosphorothoiate linkage) and 3 primers ending with VA. After 2^nd^ strand synthesis, primers ending with Adenosine were depleted by Exonuclease I digestion.

**Table 2:** **Library distribution of EXPRSS and** ***Nla*** **III-DGE multiplexed sequencing**

	Technical replicates		Biological replicates	
	EXPRSS	EXPRSS	EXPRSS	***Nla***III-DGE
**Total sequences**	7,643,302	7,959,474	12,389,113	13,030,761
**Quality filtering**	395,555	329,981	267,878	1,283,806
**Without barcode**	253,014	235,906	266,961	186,590
**1h_water_R1**	1,470,648		1,643,337	1,515,575
**1h_water_R2**	2,389,038		1,664,707	1,428,489
**1h_water_R3**	1,753,281		1,550,807	1,411,340
**1h_water_R4**	1,381,766		992,328	1,458,164
**1h_flg22_R1**		2,112,170	668,184	1,460,491
**1h_flg22_R2**		2,253,392	1,791,790	1,435,230
**1h_flg22_R3**		1,638,415	1,693,260	1,634,751
**1h_flg22_R4**		1,389,610	1,849,861	1,216,325

R1 - R4: replicates 1 to 4.

**Figure 3:**
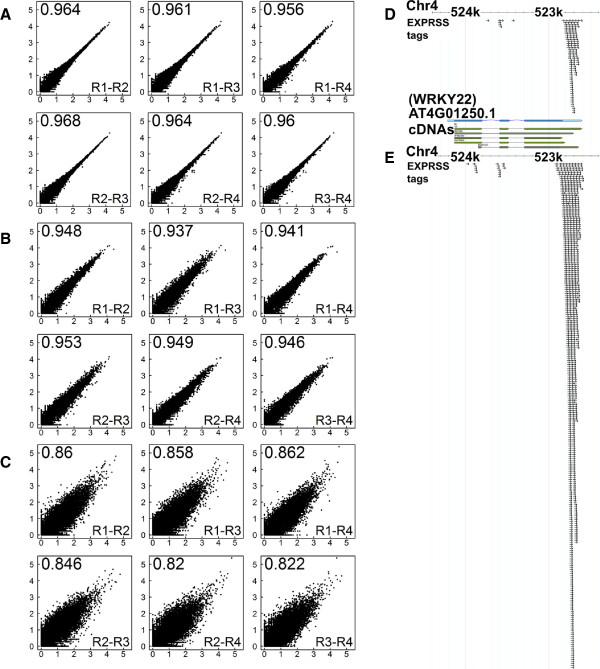
**Higher reproducibility and better dynamics of differential expression detection using EXPRSS. (A-C)** Pairwise scatter plots of gene counts from treatment replicates, expressed as tags per million in log10 scale. Correlations between four independent technical replicates of EXPRSS Tag-seq **(A)**, made from same RNA sample; four independent biological replicates of EXPRSS Tag-seq **(B)** and *Nla*III-DGE **(C)** are presented. Pearson correlation of log10 transformed tag counts per million plus 1 is depicted at left hand top corner of each comparison. Right hand bottom corner indicates replicate number depicted on X and Y-axis, respectively. **(D-E)** Example of differential expression using reads aligned (small black arrows) to *WRKY22*, a flg22 responsive gene are presented from control **(D)** and treatment **(E)**.

In order to verify that tag generation and barcoding process do not create any technical variability, 4 independent libraries were prepared from the same Arabidopsis water- and flg22- treated leaf disc RNA samples and different barcodes ligated. Eight million reads passing the Illumina quality filter were obtained for each of water and flg22-treated EXPRSS technical replicate libraries (Table 2). Pair-wise Pearson correlations of log10 transformed count data are presented in Figure 3A-C (control samples) and Additional file 1, Figure S6 (treatment samples). Very high correlations (r ≥ 0.95) between technical replicates support that the tag generation and barcoding process have not introduced any significant variability (Figure 3A and Additional file 1, Figure S6A). Similarly, higher correlation (r ≥ 0.92) was observed between biological replicates of EXPRSS Tag-seq libraries (Figure 3B - control and Additional file 1, Figure S6B - treatment), while correlations between biological replicates of *Nla*III-DGE Tag-seq libraries (r ≥ 0.82) were less than those of EXPRSS (Figure 3C and Additional file 1, Figure S6C). Fewer tag sequences were retrieved for control replicate 4 and treatment replicate 1 of EXPRSS samples (Table 2). However, the correlations between EXPRSS replicates are still very high indicating robust sampling (Figure 3A-C and Additional file 1, Figure S6). Most of the deviation for these less sequenced samples is observed for genes with very low tag frequencies compared to other replicates.

### Comparison of differential expression between EXPRSS, *Nla*III-DGE and microarray

Differential gene expression analysis was carried out using the baySeq R package [65] for both EXPRSS and *Nla*III-DGE data; genes with FDR <0.01 were classified as differentially expressed. An example of differential expression from EXPRSS data is provided in Figure 3D-E. It depicts the number of tags observed for *WRKY22*, a flg22-inducible gene encoding a WRKY transcription factor, in control and flg22- treatment data sets (Figure 3D-E, respectively). Tags observed upon flg22 treatment are ~7 times more compared to tags observed with water treatment, in line with previously published microarray analysis [41]. It is apparent that there are multiple polyadenylation sites in *WRKY22* and all of them are induced upon flg22 treatment. The distribution of tags aligning to *WRKY22* at the 3′ end is wider than expected (~150 bp). This observation is in agreement with varying lengths of cDNA, resulting from alternative polyadenylation, in the TAIR database (Figure 3E cDNA lengths). Full-length transcripts of *WRKY22* have most of their tags aligned and thus provide an example that not only illustrates the dynamics of expression differences but also supports the choice of summation of tags from alternative transcripts for each gene to identify differential expression at the gene level. Once differential expression is validated, further investigations can be carried out on alternative transcripts.

**Figure 4:**
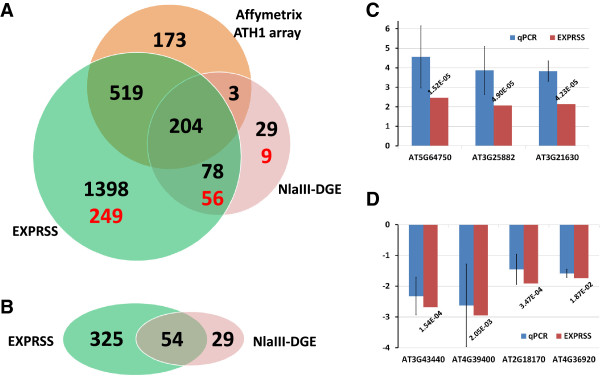
**Comparison of differential expression between EXPRSS,** ***Nla*** **III-DGE and Affymetrix ATH1 array. (A-B)** Venn diagrams showing overlap of differential expression identified from the same RNA samples (EXPRSS and *Nla*III-DGE) and similar RNA sample (ATH1 microarray). Overlap of **(A)** sense transcripts (numbers in black - genes spotted on ATH1 array and numbers in red- genes not spotted on ATH1 array) **(B)** antisense transcripts identified by EXPRSS and *Nla*III-DGE. **(C-D)** Q-PCR confirmation of differential expression observed through EXPRSS. Three up-regulated genes **(C)** and five down-regulated genes **(D)** that are found differentially expressed with EXPRSS are verified with QPCR. Error bars indicate standard deviation from three biological replicates. FDR values are provided for EXPRSS log2 fold changes.

Microarrays have been widely used for gene expression analyses. We therefore compared differential expression results derived from EXPRSS data, microarray data and *Nla*III-DGE data. EXPRSS and *Nla*III-DGE data sets were generated from the same RNA sample, while microarray data is from similar RNA samples from a previous study, hybridised to Affymetrix ATH1 chip [41]. Analysis of EXPRSS data identified differential expression in 2883 transcripts; 2504 of which were derived from sense transcripts (1066 down-regulated and 1438 up-regulated) and 379 from antisense transcripts (72 down-regulated and 307 up-regulated) (details provided in Additional file 4). A similar analysis on *Nla*III-DGE data revealed differential expression in 462 transcripts (379 sense [75 down-regulated and 304 up-regulated] and 83 antisense [19 down-regulated and 64 up-regulated] transcripts) (details provided in Additional file 5). Microarray analysis using the Rankproducts method [66] revealed differential expression in 874 probe sets (< 0.05 FDR) associated with 899 genes (370 down-regulated and 529 up-regulated) (details provided in Additional file 6). Comparisons were performed between these three methods, using only the sense gene list. Figure 4A shows the Venn diagram representing the results from a 3-way comparison. Not only does EXPRSS have a higher overlap with *Nla*III-DGE [~85% - 391 out of 462 (338 sense – hyper geometric probability 0.00 and 53 antisense – hyper geometric probability 3.51E-83)] but it also has a similar overlap with microarray data [~80% - 723 out of 899 (hypergeometric probability 0.00)], while the overlap of *Nla*III-DGE with microarray is 23% (207 out of 899). As mentioned in the introduction, one of the main advantages of Tag-seq methods is the opportunity to identify all genes expressed in a cell, unlike detecting only spotted genes on a microarray. Genes not present on the ATH1 microarray chip that are detected by EXPRSS and *Nla*III-DGE are highlighted in red in the respective section of Venn diagram (Figure 4A). To find the similarities with respect to expression changes detected between the methods compared, fold- changes of genes commonly found from each pairwise comparisons are used for correlation analysis. Correlation of fold changes from genes commonly found by EXPRSS & microarray is 0.96, EXPRSS & *Nla*III-DGE is 0.95 and microarray and *Nla*III-DGE is 0.90 (see Additional file 1, Figure S7). Correlations from expression fold-changes indicate that there is generally a good agreement between platforms and more so for EXPRSS with ATH1 microarray data and *Nla*III-DGE. The data showing higher overlap of EXPRSS data with microarray and *Nla*III-DGE, better agreement of expression changes, and increased differential expression detection, highlights the potential of EXPRSS Tag-seq as an expression profiling method.

One key observation coming from the differential expression analysis is that additional genes are identified in EXPRSS compared to the other two methods. Validation of differential expression was carried out to verify some of the genes identified from EXPRSS Tag-seq through qPCR. A set of genes spotted on the microarray or not spotted on microarray that were differentially expressed in EXPRSS was chosen for qPCR verification. Details about all the genes and their expression levels in EXPRSS and qPCR are provided in Additional file 7. Double stranded cDNA samples subjected to EXPRSS library preparation were also used for qPCR verification. Figure 4C and D verify that differential expression of selected genes could be confirmed with qPCR. Evidence from qPCR supports differential expression from the selected genes identified by EXPRSS method. However, there are 176 genes that are not differentially expressed in EXPRSS but found to be differentially expressed using microarray data. Further analysis revealed that 167 genes of these were identified in EXPRSS data and about one third of the genes had FDR between 0.01 and 0.05, suggesting that they are near the statistical threshold (Additional file 8). Most of the remaining genes had mean expression levels less than 10 TPM and high variability among replicates. Such discrepancies can thus be associated with depth of sampling and statistical techniques employed.

### Higher sensitivity of the EXPRSS method

**Figure 5:**
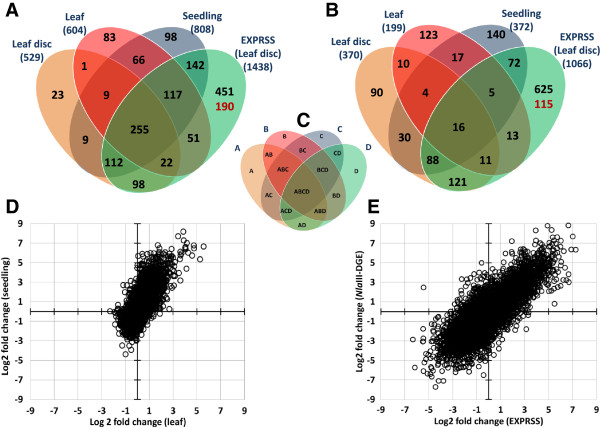
**Sensitivity of EXPRSS in differential expression detection for specific responses to treatment. (A-C)** Venn diagram representing overlap of differential expression for flg22 treatment from four different experiments (Microarray on seedling, leaf, leaf disc and EXPRSS on leaf disc) **(A)** Up-regulated genes between the four methods are compared. **(B)** Down-regulated genes between the four methods are compared. **(C)** Depiction of the overlap among the sections of Venn diagram. Number under each experiment represents number of genes differentially expressed. (Numbers in black - genes spotted on ATH1 array and numbers in red- genes not spotted on ATH1 array) **(D-E)** Scatter plots showing fold change distribution for commonly detected genes between two experiments. **(D)** Comparison of log2 fold changes between leaf and seedling microarray shows more restricted dynamics on negative scale (~ − 3) than positive scale (~ + 7). **(E)** Similar comparison of log2 fold changes between leaf disc data from EXPRSS and *Nla*III-DGE showing more even distribution (−6 to +7).

EXPRSS revealed differential expression in more genes than the other two methods and we wanted to verify if differential expression was observed for these additional genes in previous flg22 experiments. Therefore microarray data from previously published flg22 treatments of plants at various developmental stages were compared with EXPRSS Tag-seq data. ATH1 microarray data from seedlings treated with 1 μM flg22 [67], mature leaves infiltrated with 1 μM flg22 [68] and leaf discs generated from mature leaves incubated in 100 nM flg22 solution [41], were used for the analysis. Flg22 samples were compared against 1 hr water treated samples for leaf and seedling arrays and with untreated samples for leaf disc arrays. Details of differential expression are provided in Additional files 9 and 10. The Rank products method [66] was used to generate differential expression lists (< 0.05 FDR) from the 1 hr flg22 treatment microarrays and resulting lists were compared against the EXPRSS data. The outcome of these comparisons is summarised as Venn diagrams in Figure 5A and B. The majority of flg22-induced genes from various tissues are in good agreement among microarray experiments. In pair-wise comparisons of microarray data, the overlap for upregulated genes ranged from 48 to 74% (see Additional file 11). For upregulated genes EXPRSS has 92% overlap with leaf disc data (487 out of 529), 77% overlap with seedling data (626 out of 808) and 74% overlap with leaf data (445 out of 604). There is a substantial overlap of EXPRSS Tag-seq with three microarray datasets (hypergeometric probability 0.00 for three comparisons), for common as well as specific identifications. 64% of the upregulated genes identified by EXPRSS that are spotted on ATH1 chip, were found to be differentially expressed in at least one of the three microarray datasets. Although some these genes were only identified at a different developmental stage or with differing concentration of flg22, EXPRSS Tag-seq is sensitive enough to identify responding genes.

Compared to upregulated genes, the overlap of downregulated genes between three microarray experiments is less. In pair-wise comparisons of microarray data, the downregulated gene overlap ranged from 11 to 37% (see Additional file 11). However, EXPRSS profiling data has better overlap with two of the three microarray data sets. EXPRSS has 64% overlap with leaf disc data (236/370), 49% overlap with seedling data (181/372) and 23% overlap with leaf data (45/199) for downregulated genes. To understand the poor overlap of downregulated genes from three microarray experiments, fold-changes of genes commonly detected among three microarray experiments were compared pairwise. It was interesting to observe the detection dynamics is restricted for downregulation compared to upregulation (Figure 5D - scatter plot of leaf log2 fold changes against seedling log2 fold changes resulting from Rankproducts analysis). Similar analysis on the genes commonly detected between EXPRSS and *Nla*III-DGE has shown that fold-change distribution is relatively even on both positive and negative scales (Figure 5E). It appears that there is a detection bias with microarray analysis in favour of gene induction rather than down-regulation, while sequencing based methods do not show such a limitation. With such a sensitive technique available there is a tremendous opportunity to study tissue-specific as well as other response-specific genes. Higher overlap of EXPRSS Tag-seq with data from previous flg22 treatment of various tissue microarrays supports the sensitivity of the EXPRSS method.

### EXPRSS tag-seq reliability and paired-end sequencing

Expression profiling of flg22 responses in Arabidopsis ecotype Col-0 was carried out over a time course to study transcriptional regulation during flg22 triggered PTI and to validate the reliability of the EXPRSS Tag-seq. Additionally we performed a flg22 time course on mutants from three major hormonal pathways (*npr1-1* – salicylic acid [69], *jar1-1* – jasmonic acid [70] & *ein2-5* – ethylene [71]). We collected three replicates each of 7 time points of flg22 treatment (8, 15, 30, 45, 60, 120 and 180 minutes) and a water treatment control. Comparison of correlation between 3 replicates of 60 minutes flg22 samples from time course data with 4 replicates of data presented earlier, from validation of EXPRSS Tag-seq, show significantly high correlation (>0.925, see Additional file 1, Figure S8). Differential expression analysis over 60 minutes of flg22 treatment revealed ~80% (1987/2504 at < 0.01 FDR) and ~90% (2259/2504 at < 0.05 FDR) of genes found differentially expressed using 4 replicate data from EXPSS validation. The high correlation (>0.94) between log2 fold changes of 2504 genes from two experiments supports the consistency of EXPRSS Tag-seq (see Additional file 12). Additional genes found in the time course experiment could be due to comparison using a different control sample than with the validation experiment. High correlations (>0.9) have been observed between three biological replicates of 32 data points collected (4 genotypes and 8 time points; see Additional file 1, Figure S9 – S12). Samples were sequenced on different lanes of the same flow cell (wildtype and *npr1-1*), or on different flow cells from separate runs (*jar1-1* and *ein2-5*). The high correlations of replicates confirm that variation due to library preparation or sequencing lane or sequencing runs is minimal. Differential expression analysis on the time course data identified flg22-dependent gene expression changes as early as 8 minutes. Details of genes differentially expressed during the flg22 time course from four genotypes are provided in Additional file 13. Hormonal signalling mutations (*npr1-1* and *jar1-1*) responded similarly to Col-0 during flg22 treatment, while *ein2-5* appeared to show a weaker response (see Additional file 1, Figure S13). On further investigation it is evident that *ein2-5* displayed higher induction or repression of flg22-responsive genes in uninduced conditions (see Additional file 1, Figure S14). Direct comparison of expression levels during the flg22 time course from mutants and Col-0 confirmed that there are no significant differences between wild type and mutants in flg22 responsiveness, though a few genes in *ein2-5* showed a stronger response to flg22 (see Additional file 1, Figure S15).

EXPRSS Tag-seq was initially designed with Illumina single end adapters that are compatible with single read flow cells of GAII and Hiseq. We have modified EXPRSS to be compatible with the Illumina paired-end flow cell, so we can now sequence EXPRSS tag-seq samples from both ends on a GAII, a HiSeq, a HiSeq2500 and a Miseq. The second sequence read from polyadenylation junction enables verification of polyadenylation site assignments of transcripts. We have performed paired end sequencing on cDNA libraries made from temperature-shifted (28°C to 21°C – collected at 0, 6, 9 and 24 hr) samples of Arabidopsis ecotype No-0 and *slh1* leaves. We pooled eight libraries and sequencing was carried out on a Miseq and resulted in 4.6 million paired reads. As our primary focus was on the technical feasibility of the paired-end sequencing the biological relevance of this data is not further considered here. As anticipated, forward reads aligned at a defined position from the 3’ end depending on the sonication parameters (see Additional file 1, Figure S16A). For the second read, an oligodT primer comprising the Illumina P7 sequence was used as sequencing primer and 75% reads from 2^nd^ sequencing read passed the quality filtering, compared to 95% from sequencing read 1. This difference is probably due to the less complex sequencing primer used. Nevertheless, for differential expression analysis read 1 data should be sufficient and read 2 data would assist primarily in polyadenylation position assignment. The resulting paired reads enabled us to define the position of polyadenylation from selected genes (see Additional file 1, Figure S16B & S17). These data support that paired read sequencing from EXPRSS Tag-seq can be used to define the polyadenylation location of expressed genes, in addition to relative gene expression information obtained from read 1.

## Discussion

We have developed a restriction enzyme-independent Tag-seq method for expression profiling (EXPRSS) and we present evidence that it performs better than existing restriction enzyme- based (SAGE and derivative) methods. The main drawback associated with SAGE-derived NGS expression profiling methods is restriction enzymatic bias. We overcame this bias using Adaptive Focussed Acoustics from Covaris to shear cDNA and using a specific adapter, we could sequence cDNA tags from a defined position in a transcript for expression profiling. Additionally, use of sonication for fragmenting cDNA allowed us to sequence tags from all expressed genes, unlike restriction enzyme based methods, which require the enzyme recognition site in the gene to get a sequence tag. AFA shearing could be replaced with other available physical fragmentation techniques (SonicMan, Bioruptor, Hydroshear etc.), as the prerequisite is to shear cDNA to a desired size.

In principle, the EXPRSS method has the following advantages over the enzyme-based and other existing Tag-seq methods [24, 25, 43, 70–74]. EXPRSS generates one tag per transcript at a relatively defined position from the 3′ end of a gene, ensuring no length-based data transformation and enabling straightforward statistical analysis. Reverse transcription of only the 3′ ~300 bp of mRNA is required to generate a tag. Shearing using AFA, followed by gel purification, allows us to generate unbiased cDNA tags at a user-specified distance from the poly(A) site for mapping to the transcriptome. DNA shearing and DNA ligation are more high-throughput than RNA fragmentation and RNA ligation. A comparative analysis of frequency distribution of lengths of TAIR10 genes (longest transcript taken for a gene with multiple variants) against that of genes detected (1 TPM at least in 4 replicates) and differentially expressed in EXPRSS Tag-seq, revealed no preference for or against longer transcripts (see Additional file 1, Figure S18), unlike RNA-seq [26]. However, transcripts less than 250 bp are slightly depleted in detection. However, 50% of the transcripts less than 250 bp in TARI10 are pre-trna and other non-coding RNAs, suggesting EXPRSS may not lose information on protein coding genes. Tag sequences are longer than existing Tag-seq methods (~30 bp and can be up to 250 bp) thus increasing the accuracy of read mapping to reference; EXPRSS tags are directional and can distinguish sense and antisense transcripts since the strand synthesized from mRNA acts as template for sequencing, thereby providing the same strand information as that of mRNA. SMART cDNA amplification might result in amplification biases and has been shown to have reduced strand specificity [34], while EXPRSS does not use amplification for cDNA generation. If enough quantity of input is provided or enough samples are pooled, EXPRSS samples do not require any amplification before loading on to the Illumina flow cell, as they have adapters required to bind in the flow cell. Handling losses are minimal, resulting in lower variability which is an essential factor for a high-throughput method. For example in Arabidopsis, (with a mean mRNA length of ~1.5 kb based on TAIR10 transcripts) for every ~200 bp 3′-most fragment of cDNA there are 5 to 6 additional fragments resulting from shearing. These fragments act as carrier DNA until the size selection step, thereby minimizing variation in handling. EXPRSS can be carried out with minimal input of RNA and addition of a carrier DNA, at the end of the second strand cDNA reaction, reduces any losses during subsequent steps. Carrier DNA would not result in tags, as they lack sequences for amplification in the flow cell. We have successfully employed lambda phage DNA as carrier in our sample preparation without any interference. With these advantages, EXPRSS Tag-seq is an excellent method for expression profiling.

Improvements in NGS techniques have resulted in improved and increased sequence yield, thereby providing an opportunity for multiplexed tag sequencing. How should one judge sufficient depth of sampling of the library? A simulated study based on pooled SAGE data [75] indicated that a cell with a million mRNA copies, requires three million SAGE tags to identify ~90% of expressed genes. According to Zhu et al., [75] a million reads would sample ~85% of mRNA tags expressing at 1–5 copies per cell. Further evidence from recent studies [76–79] suggest that a RNA-seq sample resulting in 20–40 million reads would identify ~80% of the genes.

How do Tag-seq methods compare with whole transcriptome sequencing RNA-seq methods? RNA-seq is invaluable for annotation of a transcriptome and detection of splice variants and novel transcripts. However, RNA-seq as a tool for differential expression analysis has its drawbacks. Tag-seq methods generate one tag per transcript, mostly from a defined position, thereby avoiding transcript length biases and length-based data transformation for differential expression analysis. According to TAIR10 transcript data, the mean cDNA length of Arabidopsis is 1.5 kb. Thus, for every one fragment sequenced per transcript by EXPRSS there would be 10 ~ 150 bp fragments from RNA-seq, one could reasonably assume that 2 million reads from EXPRSS are equivalent to ~20 million reads of RNA-seq data, without transcript length and reverse transcription biases. Illumina single end sequencing on GAII provides about 35-40 M reads per lane, while a Hiseq lane provides about ~100-150 M reads. This means sequencing of only 2 RNA-seq samples per lane on GAII and 4–6 RNA-seq samples on Hiseq is required to provide sufficient depth for differential expression analysis. Multiplexing is thus more valuable with Tag-sequencing than RNA-seq. Tag-seq methods like EXPRSS therefore provide an attractive option for expression profiling with superior dynamics and reliability at substantially reduced cost.

Based on pilot experiments using four replicates each of Arabidopsis (Col-0) control and 60 minute flg22-treated leaf discs, we found that EXPRSS captures tags resulting from annotated and novel transcription units and also poly(A) transcripts from antisense strands. Read alignments indicate that cDNA is randomly sheared, as ~90% of tags are aligning around ~150 bp (±150) from the 3′ end. EXPRSS Tag-seq is highly reproducible between biological replicates and was also superior to *Nla*III-DGE Tag-seq libraries made from the same RNA samples. EXPRSS revealed expression of 26% more genes compared to *Nla*III-DGE in libraries made from the same RNA samples (16619 genes in EXPRSS against 13153 genes in *Nla*III-DGE genes with ≥1 TPM in 4 replicates). Differential expression analysis performed on absolute tag counts facilitates interpretation of expression levels. Analysis using EXPRSS has identified more than 80% of the genes found by *Nla*III-DGE and ATH1 microarray, though one could argue this is due to the higher numbers of genes found differentially expressed by EXPRSS compared to *Nla*III-DGE and ATH1 microarray. A comparative study of flg22-treated microarray data from three different developmental stages in Arabidopsis has shown that EXPRSS data had a better overlap with the three microarray data sets than the overlap observed between the three microarray data sets. Two thirds of the upregulated genes from EXPRSS (spotted on ATH1 chip) were also found differentially expressed in at least one of the three array experiments. This supports the view that EXPRSS is sensitive enough to detect response-specific differential expression.

With respect to downregulated genes, EXPRSS had good overlap with two of the three-microarray data sets; although the three-microarray data sets had poor overlap among themselves. The number of genes found downregulated, using the EXPRSS Tag-seq, was greater than those detected from other microarray experiments. Therefore, one could argue this higher overlap might be due to the higher number of genes identified as downregulated with the EXPRSS method. There appears to be a detection bias against identification of down-regulation with microarrays, explaining the poor overlap between the three microarray data sets tested. The down-regulated genes found in this study could have been previously undetected in microarray analysis. Additionally, qPCR verification of selected genes from EXPRSS data confirmed that differential expression identification is reliable. These results thus emphasize the advantages of EXPRSS Tag-seq sensitive detection and absence of hybridization related issues, unlike microarrays.

Alternative splicing is another interesting phenomenon to detect during gene expression profiling. However, finding alternative splice-junctions is computationally intricate with a tag length of ~30 bp and requires higher depth of sequencing [80]. Variable levels of expression of different alternative transcripts of a gene results in fluctuating tag densities for each variant. Li et al. [80] found that even at a tag density as high as ~20 million RNA-seq reads, only ~20% of verified alternative splice variants were discovered in the human transcriptome. Due to such insufficient tag densities, quantitative differences among alternative polyadenylation and alternative transcripts are unreliable. However, with the possibility of longer tags (~150 bp with Illumina) EXPRSS tags could be used to find alternative splice junctions, provided they occur within the last ~300 bp from a polyadenylation site.

Through EXPRSS we identified cDNA tags from regions of the genome without prior annotation. Paired end EXPRSS tag-seq could greatly improve annotation of 3′ regions and determine precise poly(A) sites for these rare transcripts. With improvements in technology the length of sequencing reads continues to increase, so that longer cDNA tags can be obtained. Longer cDNA tags not only enable us to match tags with higher confidence, but also to expression profile during host-pathogen interactions, and thus study gene expression patterns of both host and microbe. We are using such methods to address gene expression changes during Arabidopsis interactions with white rust and downy mildew.

## Conclusions

EXPRSS is a restriction enzyme-independent Tag-seq method and avoids biases of existing restriction enzyme- based (SAGE and derivative) methods. In addition to identification of expression in annotated genes, EXPRSS reveals alternative polyadenylation sites and antisense transcripts with a defined polyadenylation site. EXPRSS also identified expression in regions of the genome without prior gene annotation. EXPRSS Tag-seq provides sensitive and reliable gene expression data and enables high-throughput expression profiling. Sequencing technologies continue to improve and costs of sequencing continue to decline. However, as noted by Sboner et al., [81], even though the cost of sequencing has reduced, the cost of experimentation hasn’t. It is therefore vital that not only sample preparation and sequencing costs are reduced, but the downstream analysis should also be relatively simple. The EXPRSS method by all these criteria is highly suitable for cost-effective expression profiling of large numbers of mRNA samples.

## Materials and methods

### Plant material and flg22 treatment

*Arabidopsis thaliana* (Col-0, *npr1-1*, *jar1-1* and *ein2-5*) plants were grown under short-day conditions (8 hr light/16 hr dark cycles). No-0 and *slh1* plants were grown in short day conditions (10 h light / 14 h dark) for 4 weeks at 28°C after germination on MS plate. Leaf discs from 5-week-old Col-0, *npr1-1*, *jar1-1* and *ein2-5* plants were prepared 2 hours after start of light period and vacuum-infiltrated with water for 1 min. Leaf discs were placed in water in 50 mm petri dishes and left in the same growing conditions for ~20 h. A day after leaf disc generation the water in the petri dishes was replaced with water or 100 nM flg22 and samples were harvested 1 h after incubation. For flg22 time course experiment samples were harvested after 8 minutes incubation in water and 8, 15, 30, 45, 60, 120 & 180 minutes incubation in 100 nM flg22. Four weeks old No-0 and *slh1* plants grown at 28°C were transferred to 21 °C growth chamber at the beginning of the light cycle and samples were harvested at 0 h, 6 h, 9 h and 24 h after transfer.

### RNA preparation

Total RNA was extracted using the TRI reagent (Sigma) and 1-Bromo-3-chloropropane (Sigma), as per manufacturer’s guidelines. RNA was precipitated with half volume of isopropanol and half volume of high salt precipitation buffer (0.8 M sodium citrate and 1.2 M sodium chloride). RNA samples were treated with DNaseI (Roche) according to the manufacturer’s recommendation and phenol/chloroform extracted and ethanol precipitated.

### Q-RT-PCR

For quantitative Reverse Transcription PCR, RNA samples were reverse-transcribed into complementary DNA (cDNA) using SuperscriptII reverse transcriptase (Invitrogen). The cDNA was used to quantify gene expression using a SYBR Green quantitative PCR kit (Sigma) and gene-specific primers (see Additional file 2). Themocycling and intensity detection was carried out with Chromo4 system on MJ Research Thermal cycler and data extracted using Opticon Monitor software.

### Tag-seq library preparation and sequencing

#### EXPRSS

Typically 5 μg of total RNA was used to generate first strand cDNA using a oligo (dT) primer comprising P7 sequence of Ilumina flow cell. Double strand cDNA is synthesized as described previously [38]. Purified cDNA is subjected to Covaris shearing (parameters: Intensity – 5, Duty cycle – 20%, Cycles/Burst – 200, Duration – 90 seconds). End repairing and A-tailing of sheared cDNA is carried out as described by Illumina. Y-shaped adapters are ligated to A-tailed DNA and subjected to size selection on 1X TAE agarose gel. The gel-extracted library is PCR enriched and quantified using qPCR with previously sequenced similar size range Illumina library.

#### *Nla*III-DGE

Library preparation was carried out with 5 μg of total RNA as mentioned previously [43]. In our modified protocol, ligation of biotinylated Adapter1, harbouring methylated adenine, was carried out in the presence of *Nla*III and T4 DNA ligase at 37°C as described previously [44]. After Adapter2 (which contains barcode) ligation, streptavidin magnetic beads (Promega) were used to capture ligated constructs and which were taken forward for PCR enrichment as in the default protocol. Detailed protocols for EXPRSS and *Nla*III-DGE are provided in Additional file 2. Both EXPRSS and *Nla*III-DGE libraries are sequenced on Illumina Genome Analyzer II.

### Bioinformatics analysis and data processing

Illumina sequence library is quality filtered using FASTX Toolkit 0.0.13 with parameters -q20 and -p50 [64] and reads containing “N” are discarded and read qualities are converted from Illumina fastq to sanger fastq format. EXPRSS libraries are separated using perfect match to the barcode. For *Nla*III-DGE, FASTX Toolkit fastx_clipper is used to trim 3′ adapter sequence (parameters used -a TCGTATGCCGTCTTC -l 18 –c) and libraries are separated using barcodes allowing up to 1 mismatch. Each sub-library is quality filtered (−q20 and -p50) and artefact filtered using FASTX-toolkit. For *Nla*III-DGE libraries *Nla*III recognition site (CATG) was added at 5′ end of each tag sequence and “FFFF” as fastq quality. Quality filtered library was aligned to the Arabidopsis thaliana Col-0 genome sequences (TAIR10) [48] using Bowtie version 0.12.8 (−a -m 10 --best --strata) [49] and selected reads with up to 10 reportable alignments. Unaligned reads from previous step are used to align to transcript sequences of Arabidopsis [61] using Bowtie version 0.12.8 (−a -m 100 --best --strata).

Tag to gene association is carried out using following considerations. Reads aligning in sense orientation with in each gene limits are assigned to that gene. A read aligning to all splice variant of a gene is counted once, and the reads uniquely aligning to various splice variants of a gene are pooled. Reads aligning to genes with overlapping gene limits are split equally between them. Reads aligning to more than 10 genes are discarded. Reads aligning to up to 10 genes are split equally between them. Reads aligning on the anti-sense strand of any gene are tested for if the read falls within 500 bp from 3′ end of another gene in sense direction. Otherwise reads are assigned as antisense tags for the specific gene (see Additional file 1, Figure S19). The tag assignment process was implemented in perl and scripts are available on request. Our analysis pipeline modules for implementation in Galaxy web-based research platform are provided in Additional file 3.

Differential expression analysis was performed using the R statistical language version 2.15.3 [82] with the Bioconductor [83] package baySeq version 1.12.0 [65] using 10000 iterations to estimate empirical distribution on the parameters of the Negative Binomial distribution. The Multi-Experiment Viewer software from the TIGR website [84] was used to cluster similarly expressed genes using Hierarchical clustering [85].

Eight samples were pooled and sequenced at 151 bp paired end reads in a Miseq from each end of the EXPRSS library. The insert size of the library was > = 150 bp and also to be comparable with other runs of this manuscript sequence reads were truncated to 36 bp and quality filtered and used for downstream analysis. Paired end reads were aligned to Arabidopsis genome sequence (TAIR10) using Burrows-Wheeler Aligner (BWA)[86] sampe with default parameters. Generated Sequence Alignment/Map (SAM) format files are converted to sorted bam files and indexed using SAMtools [87]. Sorted bam files with index are loaded on to Integrative Genomics Viewer (IGV) v2.3.8 [88] for visualization of paired reads.

### Microarray data analysis

Microarray data cel files from flg22 treatment on the seedlings (NASCARRAYS-409) [67], mature leaves (NASCARRAYS-122) [68] and leaf discs generated from mature leaves (GEO accession number GSE17479) [41] were used. Data analysis was performed using the R statistical language with the Bioconductor packages limma [89] and affy [90]. Robust multiarray average background-corrected, quantile normalized, and log-transformed perfect match only expression values were obtained using medianpolish summary method [91]. Differentially expressed genes were identified using the rank products method with a false discovery rate of <0.05 [66].

### Availability of supporting data

The sequence data discussed in this publication have been deposited in NCBI’s Gene Expression Omnibus [92] and are accessible through GEO Series accession number GSE51721.

## Electronic supplementary material

Additional file 1: **Supplemental Figures.** **Figure S1.** Modified *Nla*III-DGE Tag-seq protocol. **Figure S2.** Transcription at rRNA loci observed with EXPRSS and *Nla*III-DGE tag sequencing. **Figure S3.** Correlation between sense and anti-sense transcript expression. **Figure S4.** Novel transcription detection using EXPRSS Tag-seq. **Figure S5.** Cumulative frequency distribution multi-matching reads. **Figure S6.** Pair-wise scatter plots of gene counts from flg22 treated replicates. **Figure S7.** Pair-wise correlation of fold changes between three methods tested. **Figure S8.** Pair-wise scatter plots of gene counts from 60 minutes flg22 treatment replicates from two independent experiments. **Figure S9.** Pair-wise scatter plots of gene counts from Col-0 flg22 time course replicates. **Figure S10.** Pair-wise scatter plots of gene counts from *npr1-1* flg22 time course replicates. **Figure S11.** Pair-wise scatter plots of gene counts from *jar1-1* flg22 time course replicates. **Figure S12.** Pair-wise scatter plots of gene counts from *ein2-5* flg22 time course replicates. **Figure S13.** Hierarchical clustering of genes differentially expressed during flg22 time course of four genotypes. **Figure S14.** Heat maps of log2 fold changes from all data points of genes that are differentially expressed at least from one time point of flg22 time course. **Figure S15.** Hierarchical clustering of genes differentially expressed during flg22 time course of four genotypes compared to Col-0. **Figure S16.** Frequency distribution of Read1 and Read2 from paired end sequencing. **Figure S17.** Examples showing Read1 and Read2 from paired end sequencing. **Figure S18.** Length distribution of genes detected in EXPRSS. **Figure S19.** Cartoon depicting tag assignment to genes. (PDF 8 MB)

Additional file 2: **Detailed protocols of EXPRSS and NlaIII-DGE and information of primers used.** (PDF 142 KB)

Additional file 3: **Zip archive of the analysis tools designed to import in to Galaxy web tool.** (ZIP 2 MB)

Additional file 4: **Details of genes differentially expressed in EXPRSS Tag-seq.** (XLSX 7 MB)

Additional file 5: **Details of genes differentially expressed in NlaIII-DGE Tag-seq.** (XLSX 6 MB)

Additional file 6: **Details of genes differentially expressed in ATH1 array flg22 treatment of leaf discs.** (XLSX 143 KB)

Additional file 7: **Details from EXPRSS Tag-seq, for genes used for qPCR verification.** (XLSX 45 KB)

Additional file 8: **Details of genes differentially expressed in ATH1 array (leaf disc) but not in EXPRSS Tag-seq.** (XLSX 81 KB)

Additional file 9: **Details of genes differentially expressed in ATH1 array flg22 treatment of leaf.** (XLSX 132 KB)

Additional file 10: **Details of genes differentially expressed in ATH1 array flg22 treatment of seedlings.** (XLSX 169 KB)

Additional file 11: **Overlap among three flg22 ATH1 array differential expression represented as pair-wise comparison.** (XLSX 12 KB)

Additional file 12: **Locus details, log 2 fold change values and FDR of differentially expressed genes at 60 minutes of flg22 treatment from two independent experiments.** (XLSX 1 MB)

Additional file 13: **Details of genes and log 2 fold change values of differentially expressed during flg22 time course in Col-0,** ***npr1-1,*** ***jar1-1*** **and** ***ein2-5.*** (XLSX 8 MB)

## Abbreviations

AFA: Adaptive focussed acoustics
SAGE: Serial analysis of gene expression
DGE: Digital gene expression
EST: Expressed sequence tag
RNA-seq: Whole transcriptome sequencing
Tag-seq: cDNA tag sequencing
TPM: Tags per million
FDR: False-discovery rate
DE: Differential expression.
