# Supplementary material for: EXPRSS: an Illumina based high-throughput expression-profiling method to reveal transcriptional dynamics
Source: BMC Genomics. 2014 May 6;15(1):341. doi: 10.1186/1471-2164-15-341 (PMC4035070; doi:10.1186/1471-2164-15-341)
Supplement: Supplementary file 1 — Additional file 1: Supplemental Figures. Figure S1. Modified NlaIII-DGE Tag-seq protocol. Figure S2. Transcription at rRNA loci observed with EXPRSS and NlaIII-DGE tag sequencing. Figure S3. Correlation between sense and anti-sense transcript expression. Figure S4. Novel transcription detection using EXPRSS Tag-seq. Figure S5. Cumulative frequency distribution multi-matching reads. Figure S6. Pair-wise scatter plots of gene counts from flg22 treated replicates. Figure S7. Pair-wise correlation of fold changes between three methods tested. Figure S8. Pair-wise scatter plots of gene counts from 60 minutes flg22 treatment replicates from two independent experiments. Figure S9. Pair-wise scatter plots of gene counts from Col-0 flg22 time course replicates. Figure S10. Pair-wise scatter plots of gene counts from npr1-1 flg22 time course replicates. Figure S11. Pair-wise scatter plots of gene counts from jar1-1 flg22 time course replicates. Figure S12. Pair-wise scatter plots of gene counts from ein2-5 flg22 time course replicates. Figure S13. Hierarchical clustering of genes differentially expressed during flg22 time course of four genotypes. Figure S14. Heat maps of log2 fold changes from all data points of genes that are differentially expressed at least from one time point of flg22 time course. Figure S15. Hierarchical clustering of genes differentially expressed during flg22 time course of four genotypes compared to Col-0. Figure S16. Frequency distribution of Read1 and Read2 from paired end sequencing. Figure S17. Examples showing Read1 and Read2 from paired end sequencing. Figure S18. Length distribution of genes detected in EXPRSS. Figure S19. Cartoon depicting tag assignment to genes. (PDF 8 MB) [file 12864_2013_6041_MOESM1_ESM.pdf]

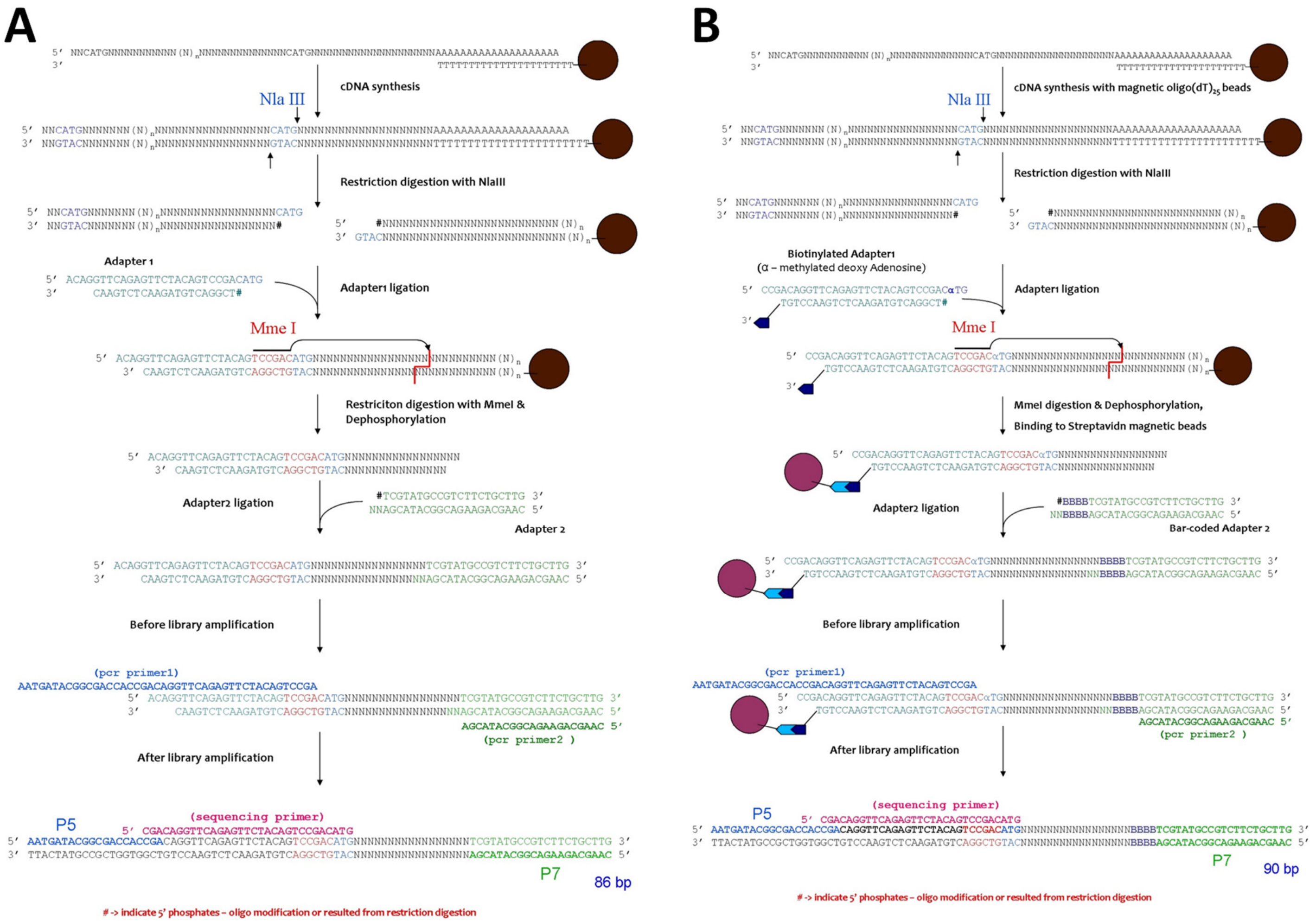

Figure S1: Modified *Nla*III-DGE Tag-seq protocol

(A) Protocol of Illumina Genome Analyzer *Nla*III-DGE (B) Modified *Nla*III-DGE protocol, customized for multiplexing and improved cDNA tag sequence generation (Modifications are – biotinylated and methylated Adapter1, barcoded Adapter2 "BBBB" 4 base barcode)

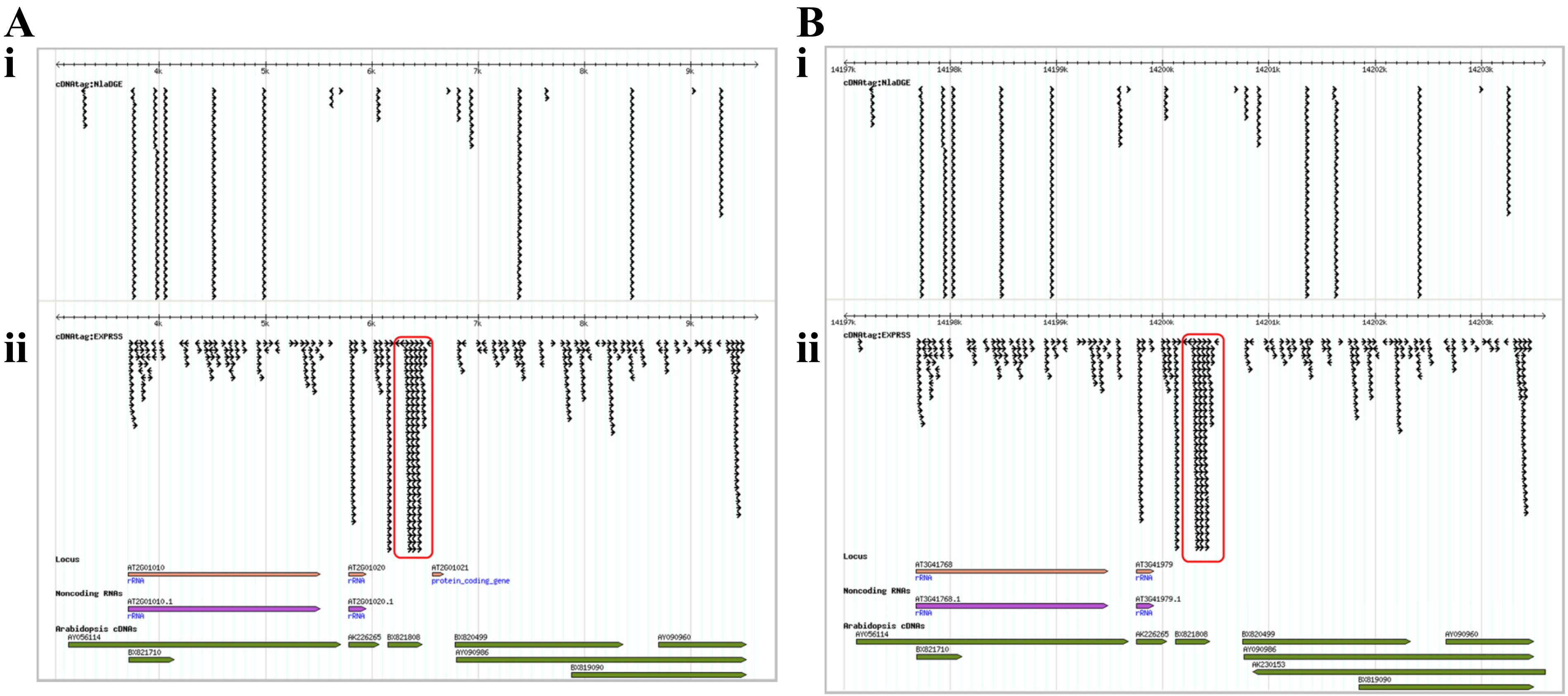

Figure S2: Transcription at rRNA loci observed with EXPRSS and *Nla*III-DGE tag sequencing

Arabidopsis genome browser (TAIR10) snapshot, showing rRNA encoding transcripts from chromosome 2 (A) and chromosome 3 (B). *Nla*III-DGE (A i & B i) and EXPRSS (A ii & B ii) Tag-seq reads (small black arrows), aligning to rRNA loci on chromosome 2 and 3 are presented. Major part of EXPRSS reads (encased in red box) are observed downstream of AT2G01020 and AT3G41768 with cDNA support (green block arrow – BX821808). No tags were detected in *Nla*III-DGE from this transcript, due to lack of *Nla*III restriction site. Tag alignment snapshots for EXPRSS and NlaIII-DGE are cropped to show top part of alignments for clarity.

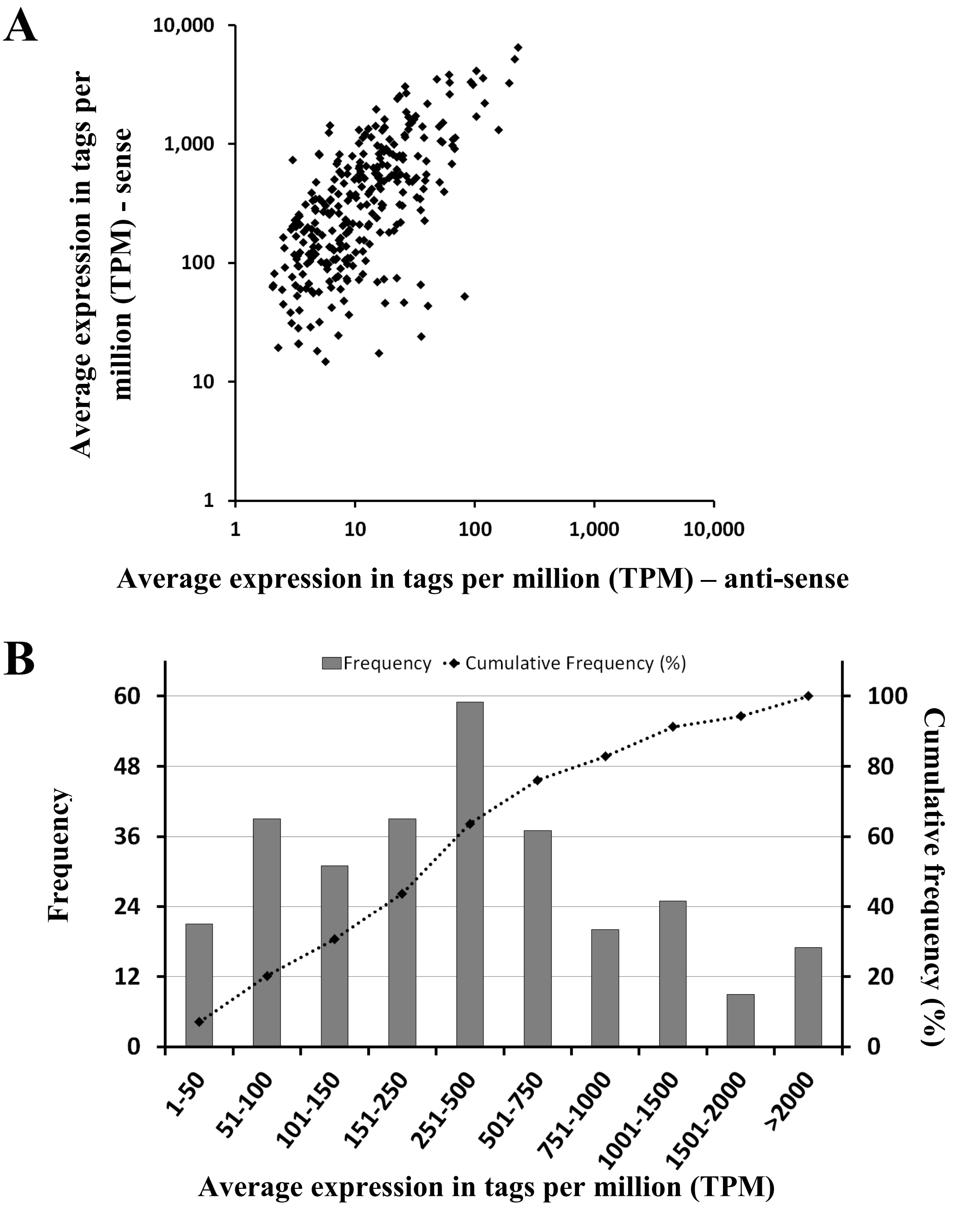

Figure S3: Correlation between sense and anti-sense transcript expression
Genes that are differentially expressed for their sense and antisense transcripts are selected (n=297). Average expression (in tags per million) was estimated for each sense and anti-sense pair from treatment replicates for 263 upregulated and from control replicates for 34 downregulated genes. (A) Mean expression of sense (Y-axis) and respective anti-sense (X-axis) transcripts are plotted pairwise (Pearson correlation 0.76). Apart from a few genes on the diagonal, especially transcripts with high expression, the majority of the genes are distributed on either side of the diagonal. (B) The sense transcripts of 297 genes are grouped based on their average expression and their frequency and cumulative frequency are plotted.

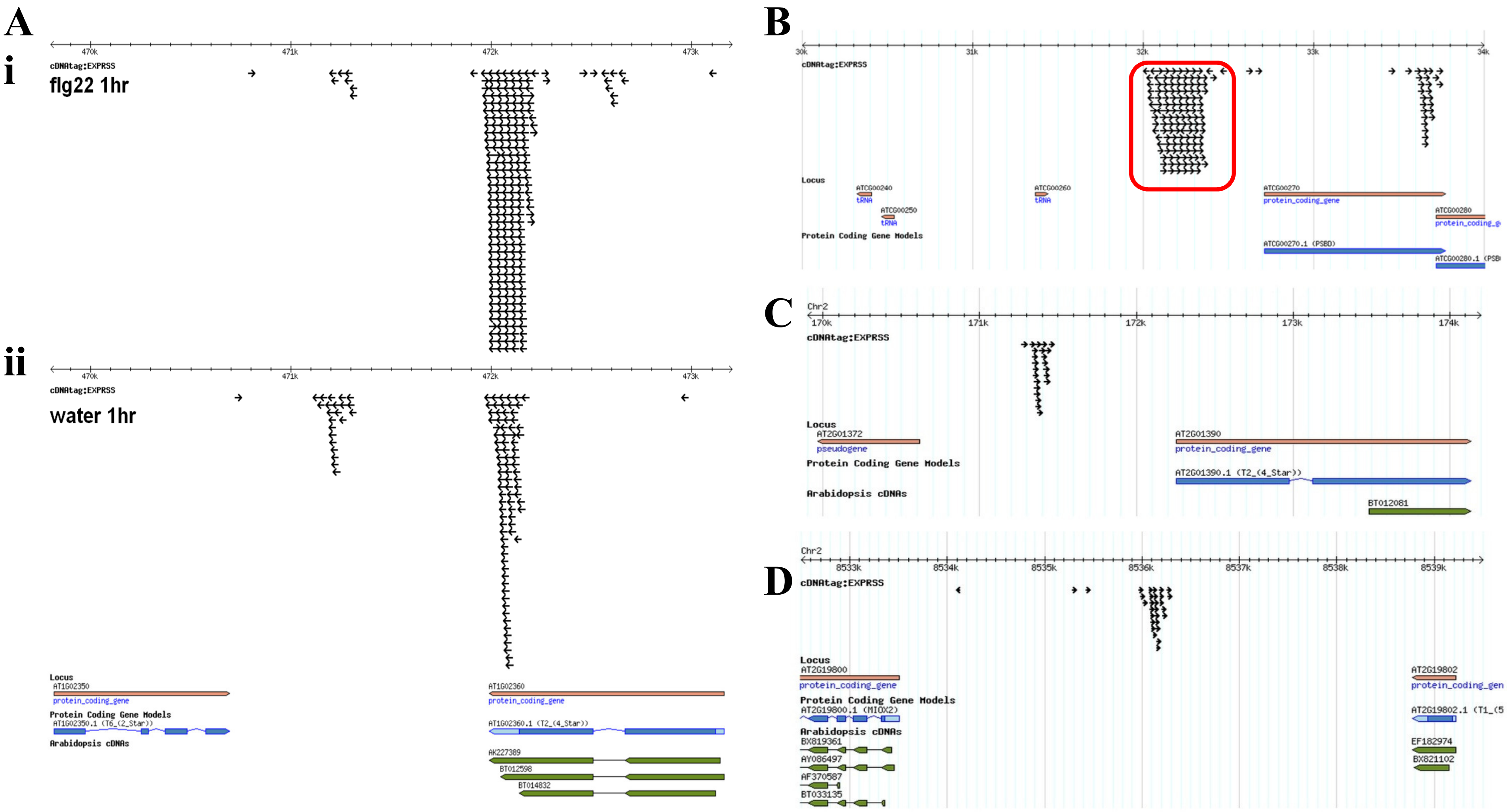

Figure S4: Novel transcription detection using EXPRSS Tag-seq.

Regions of the genome showing EXPRSS tags without prior gene annotation are presented. (A) AT1G02360 showing flg22 dependent upregulation (32 folds), while a novel transcript downstream of it shows 4 fold downregulation (A i) flg22 snapshot and (A ii) water sample snapshot. (B) A novel cholorplastic transcript upstream of ATCG00270 (reads highlighted in red box), has shown very high and comparable expression in all samples sequenced. (C-D) Unassigned alignments are pooled from all replicates of flg22 treatment samples (~150,000 from 4 replicates) and presented on the Arabidopsis TAIR10 genome browser showing evidence for novel transcription. Tag alignment snapshots for Ai and B are cropped to show top part of alignments for clarity

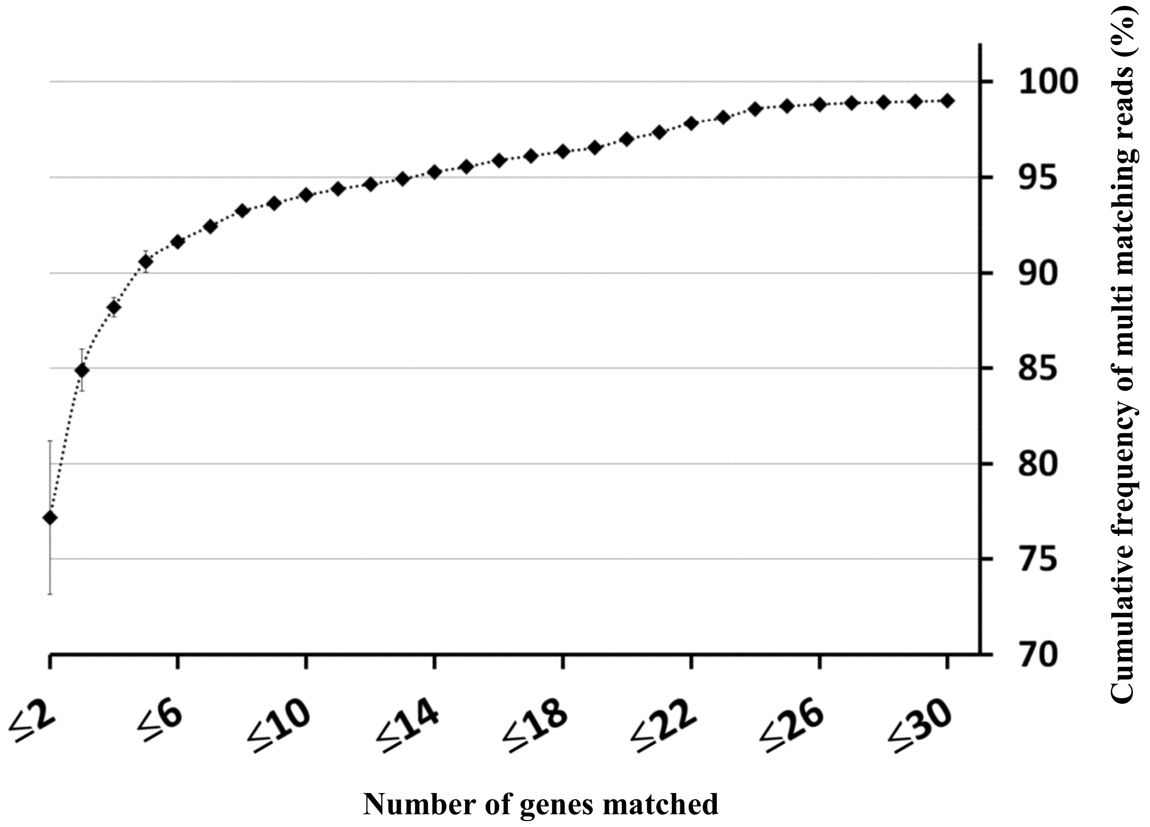

Figure S5: Cumulative frequency distribution of multi-matching reads
Reads matching to more than one gene are counted and their cumulative frequency distribution as increments of 1 gene, are plotted against number of genes matched.

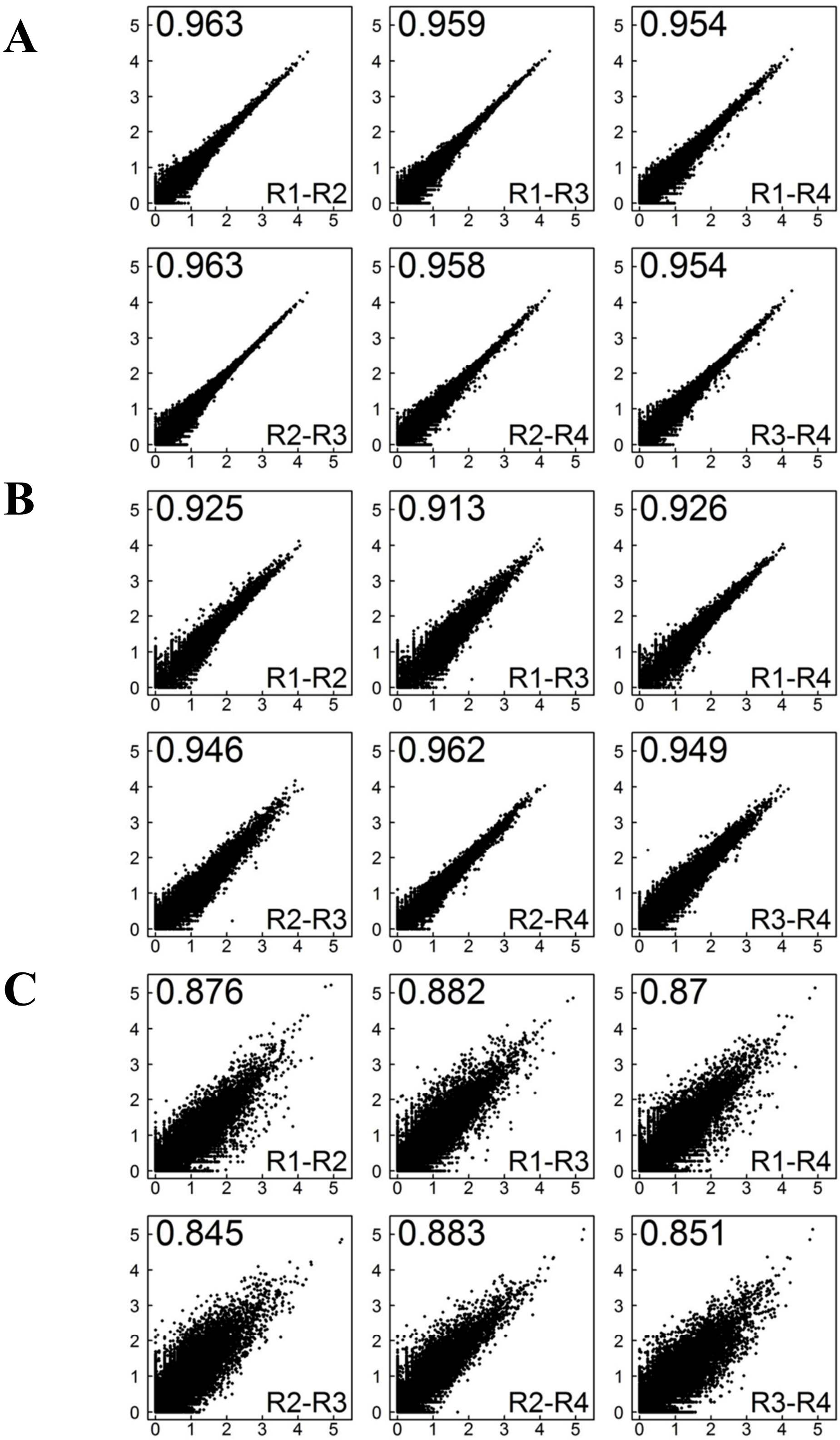

Figure S6: Pair-wise scatter plots of gene counts from flg22 treated replicates

Pair-wise correlation of technical (A) and biological (B) replicates of EXPRSS and biological (C) replicates of *Nla*III-DGE of flg22 treatment samples are presented. Top left hand corner of each square shows Pearson correlation of $\log_{10}$ transformed tag counts per million plus 1 of the pair in comparison. R1 to R4 – replicate 1 to 4.

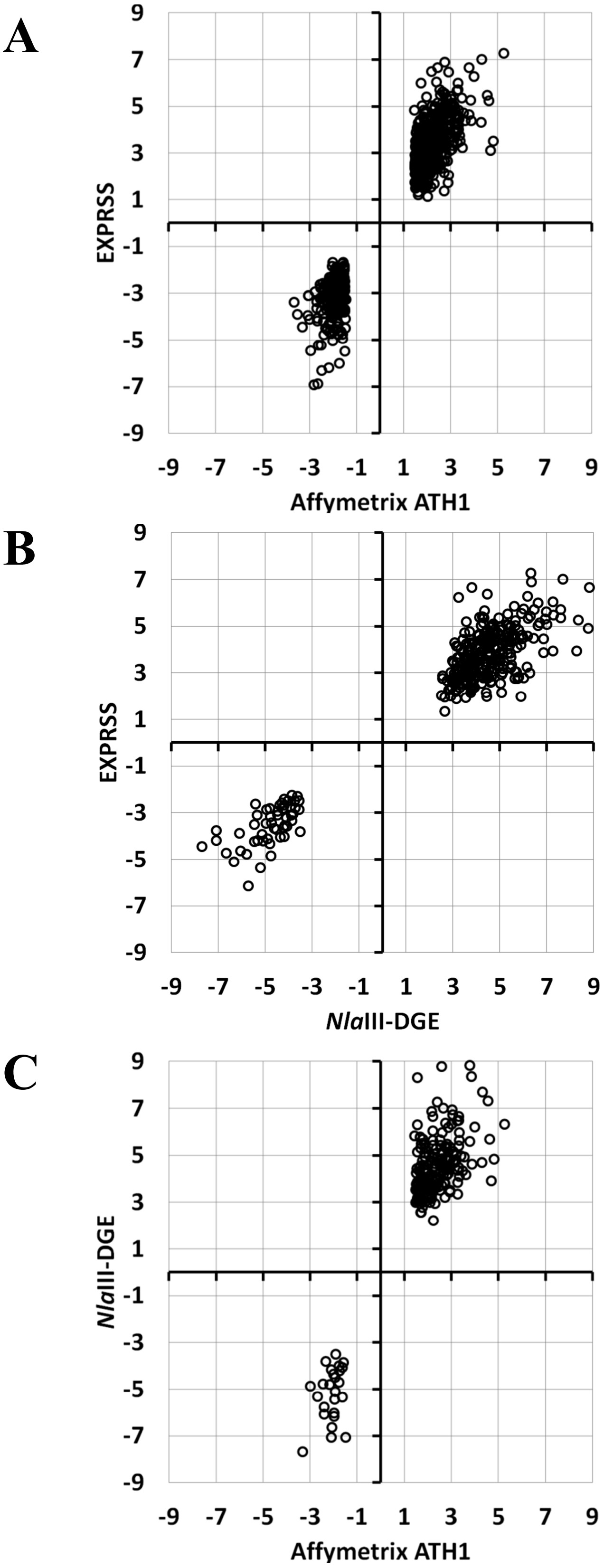

Figure S7: Pair-wise correlation of fold changes between three methods tested

Log2 fold changes of genes overlapping between respective methods are used for scatter plots. (A) EXPRSS vs Affymetrix ATH1 array (n=723 and r=0.96); (B) EXPRSS vs *Nla*III-DGE (n=338 and r=0.95) and (C) *Nla*III-DGE vs Affymetrix ATH1 array (n=207 and r=0.90). n – Number of genes and r – Pearson correlation

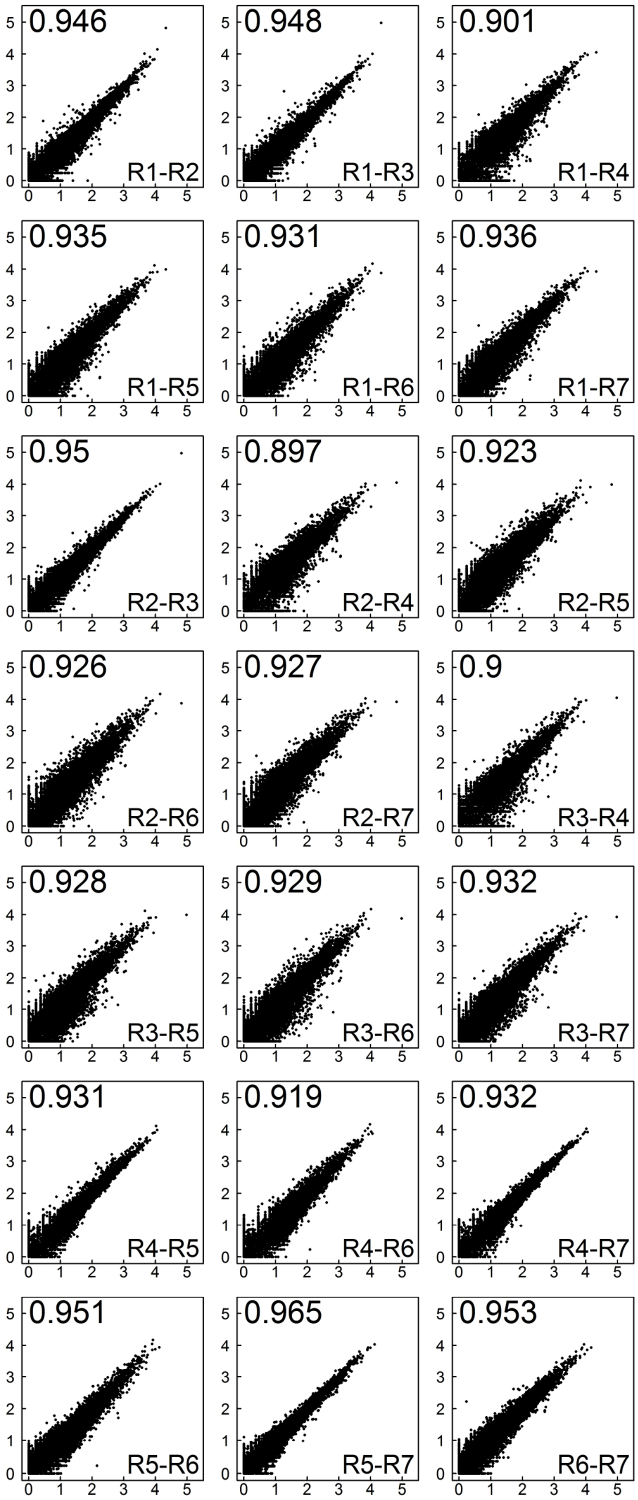

Figure S8. Pair-wise scatter plots of gene counts from 60 minutes flg22 treatment replicates from two independent experiments

Pair-wise correlations of biological replicates from two independent experiments are presented for 60 min flg22 treated Col-0 samples. Top left hand corner of each square shows Pearson correlation of $\log_{10}$ transformed tag counts per million plus 1 of the pair in comparison. The correlation among replicates from within or between independent experiments is high. R1 to R4 – four biological replicates from EXPRSS Tag-seq validation experiment and R5 to R7 three biological replicates from Col-0 flg22 time course experiment.

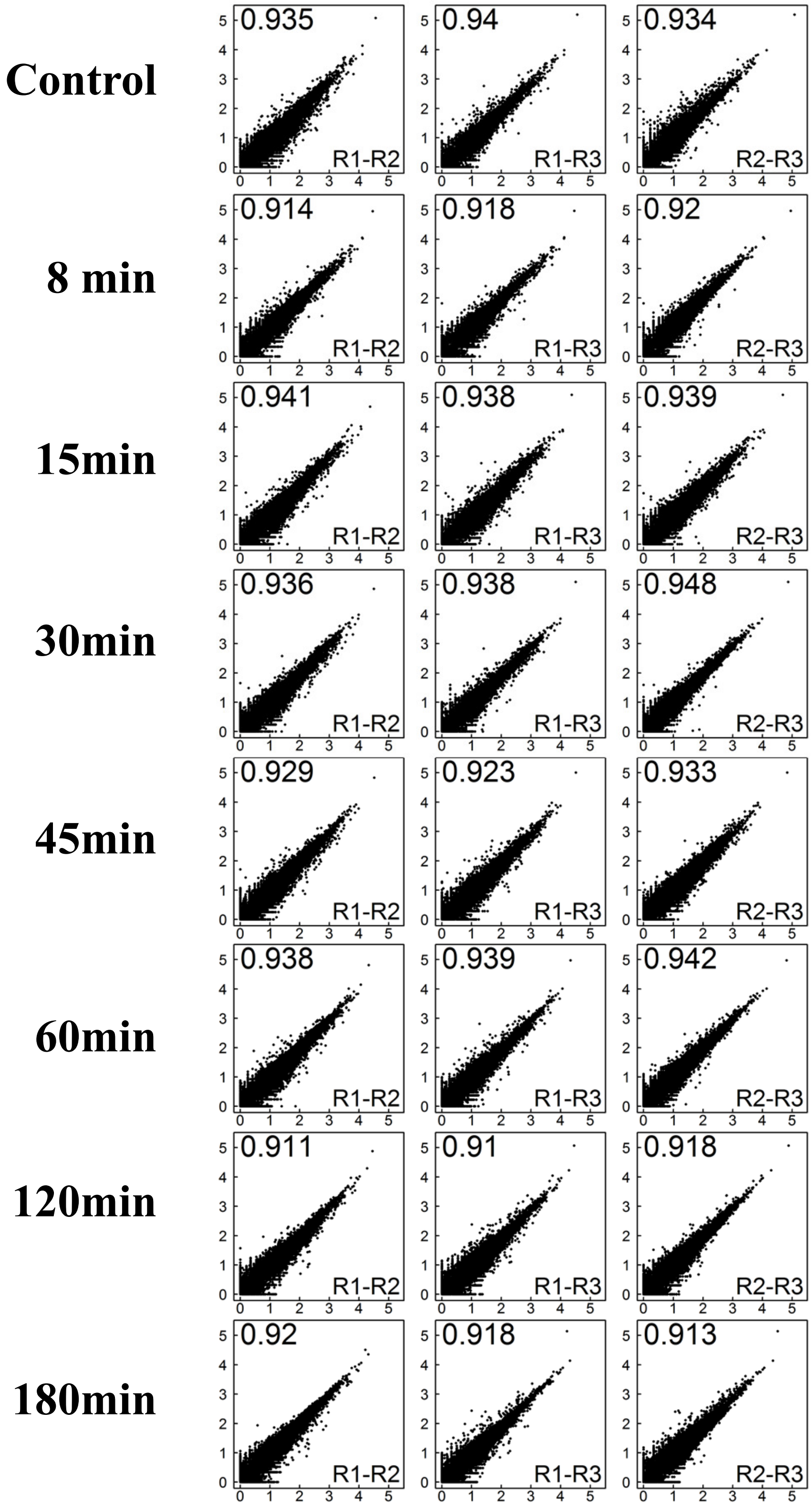

Figure S9. Pair-wise scatter plots of gene counts from Col-0 flg22 time course replicates
Pair-wise correlation of each three biological replicates of Col-0 flg22 treatment time course samples are presented. Top left hand corner of each square shows Pearson correlation of $\log_{10}$ transformed tag counts per million plus 1 of the pair in comparison. R1 to R3 – three biological replicates.

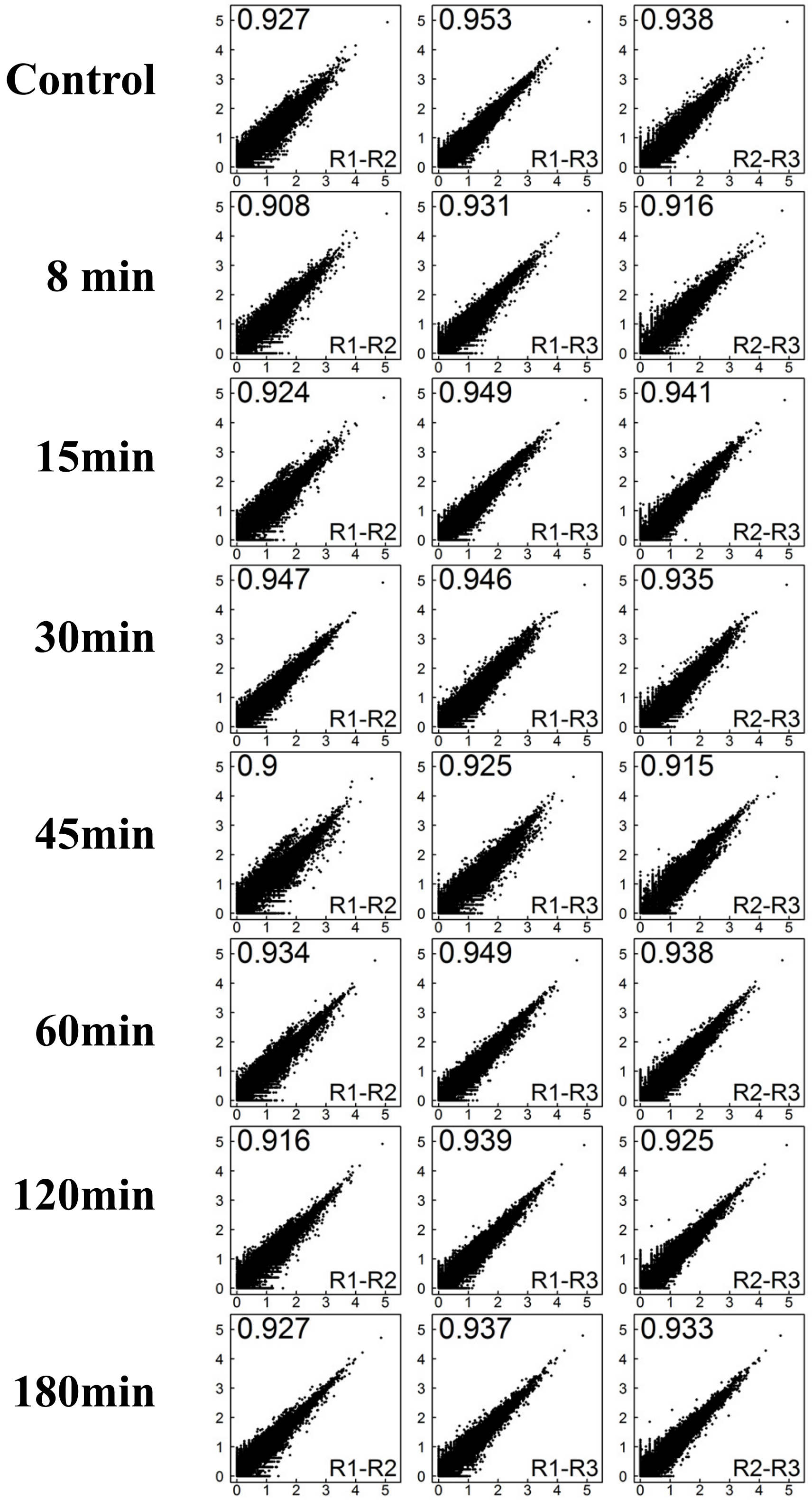

Figure S10. Pair-wise scatter plots of gene counts from *npr1-1* flg22 time course replicates

Pair-wise correlation of each three biological replicates of *npr1-1* flg22 treatment time course samples are presented. Top left hand corner of each square shows Pearson correlation of $\log_{10}$ transformed tag counts per million plus 1 of the pair in comparison. R1 to R3 – three biological replicates.

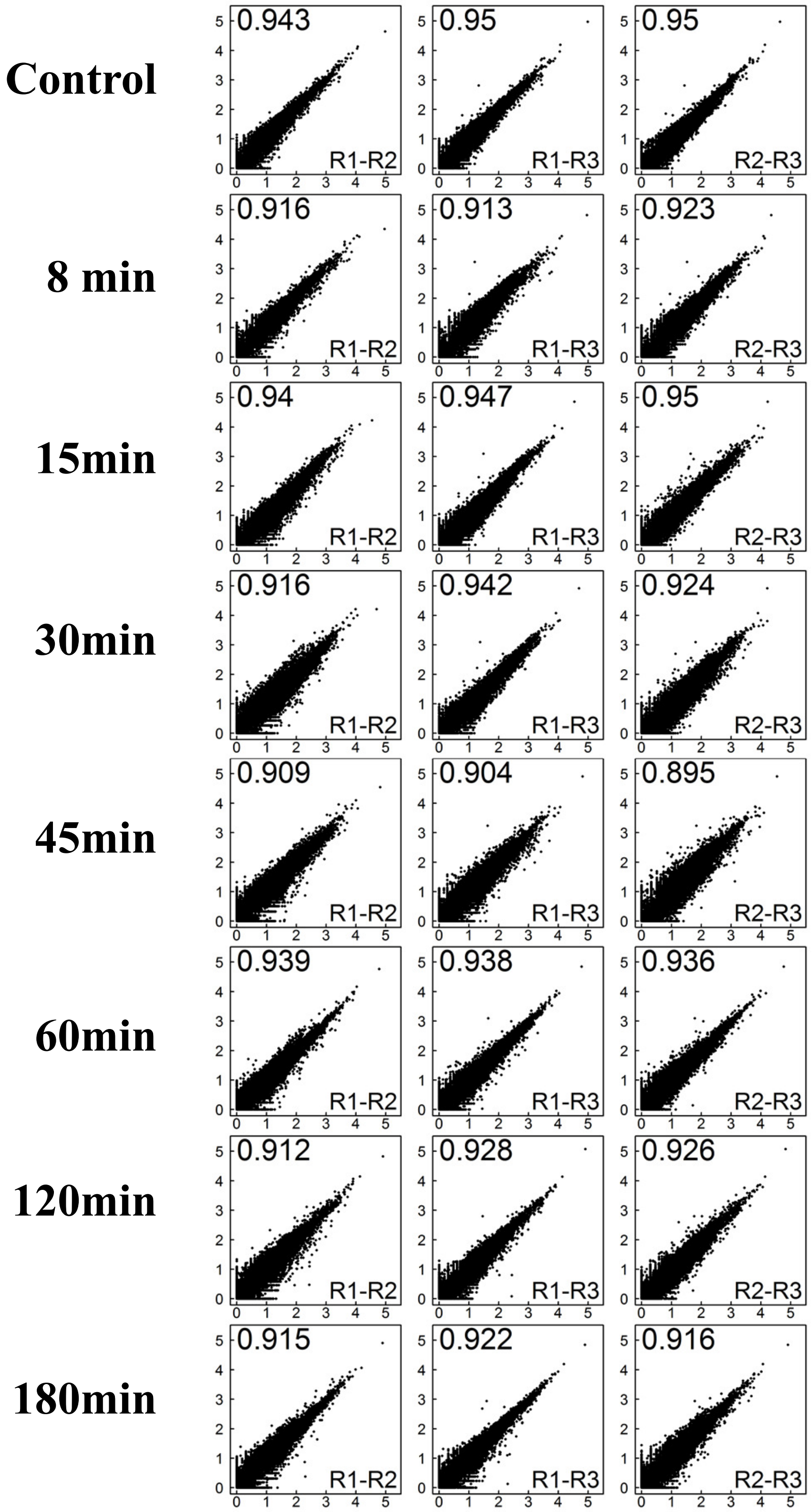

Figure S11. Pair-wise scatter plots of gene counts from *jar1-1* flg22 time course replicates
Pair-wise correlation of each three biological replicates of *jar1-1* flg22 treatment time course samples are presented. Top left hand corner of each square shows Pearson correlation of $\log_{10}$ transformed tag counts per million plus 1 of the pair in comparison. R1 to R3 – three biological replicates.

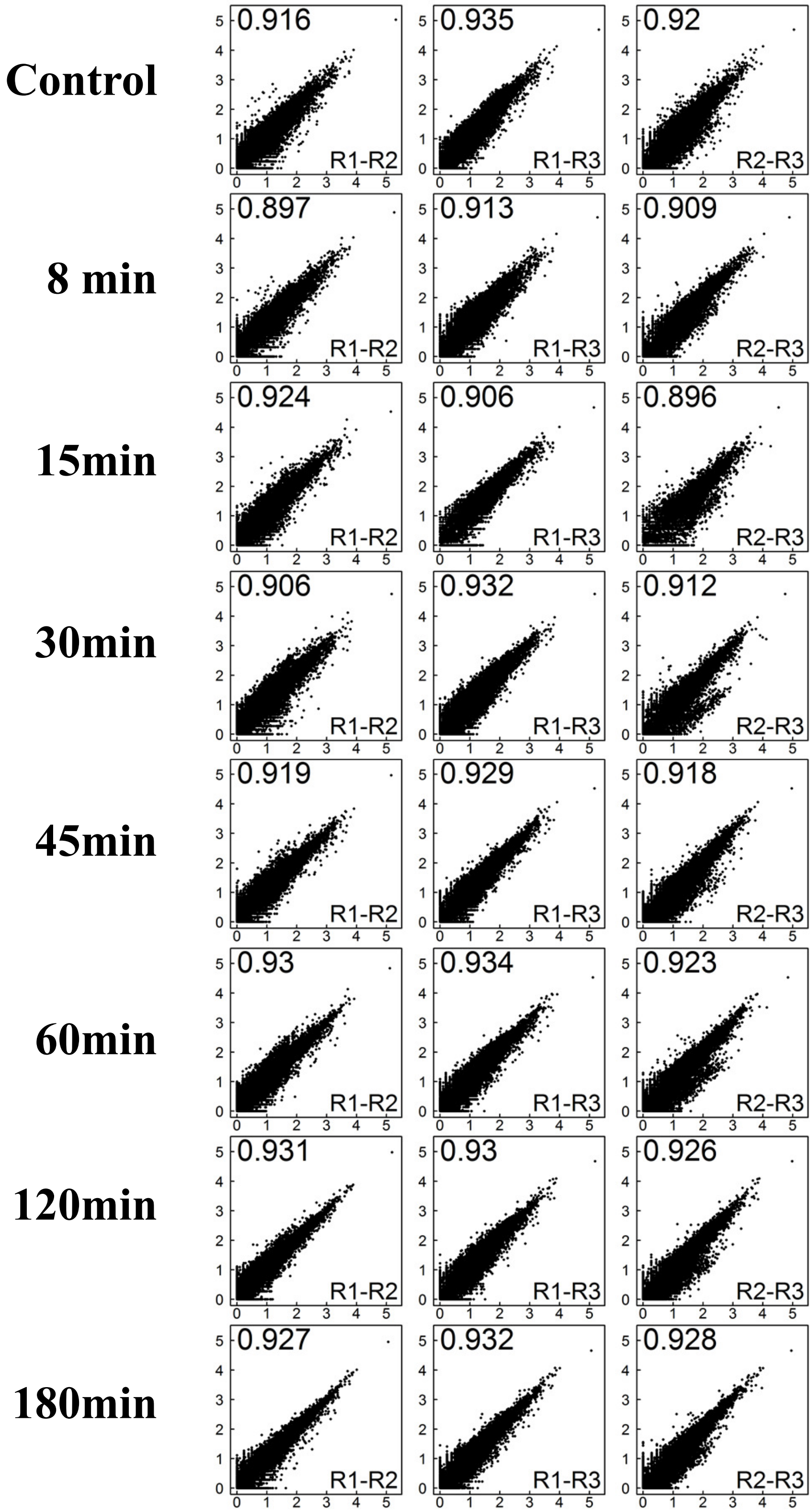

Figure S12. Pair-wise scatter plots of gene counts from *ein2-5* flg22 time course replicates
Pair-wise correlation of each three biological replicates of *ein2-5* flg22 treatment time course samples are presented. Top left hand corner of each square shows Pearson correlation of $\log_{10}$ transformed tag counts per million plus 1 of the pair in comparison. R1 to R3 – three biological replicates.

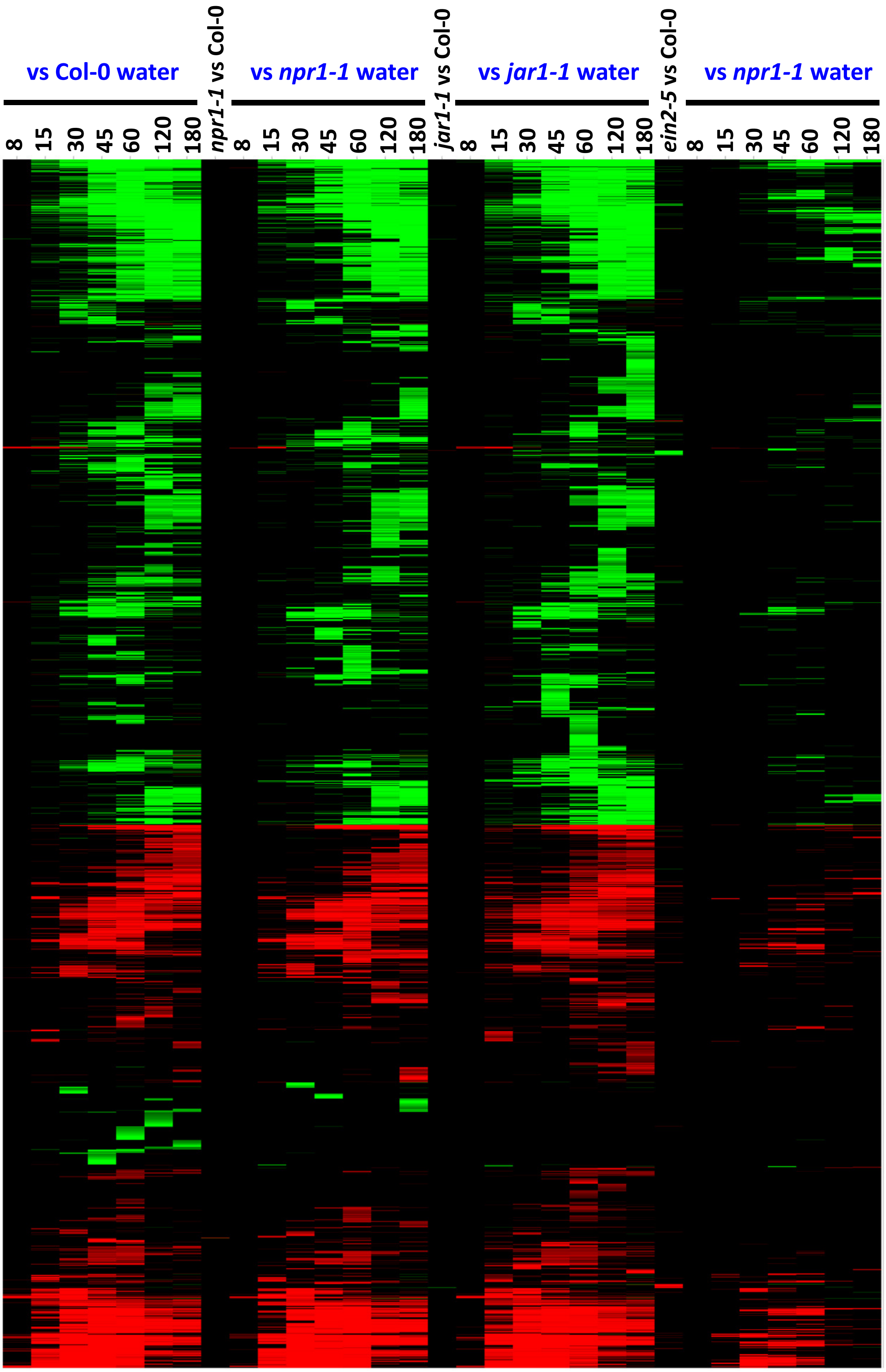

Figure S13. Hierarchical clustering of genes differentially expressed during flg22 time course of four genotypes

Heat map showing hierarchically clustered log_2 fold changes of genes differentially expressed during flg22 time course (n=9282) from the four genotypes (Col-0, *npr1-1*, *jar1-1* and *ein2-5*) studied. The four genotypes have responded to flg22, while response from *ein2-5* appears to be reduced. For each genotype flg22 treated samples are compared to water treated sample of respective genotype.

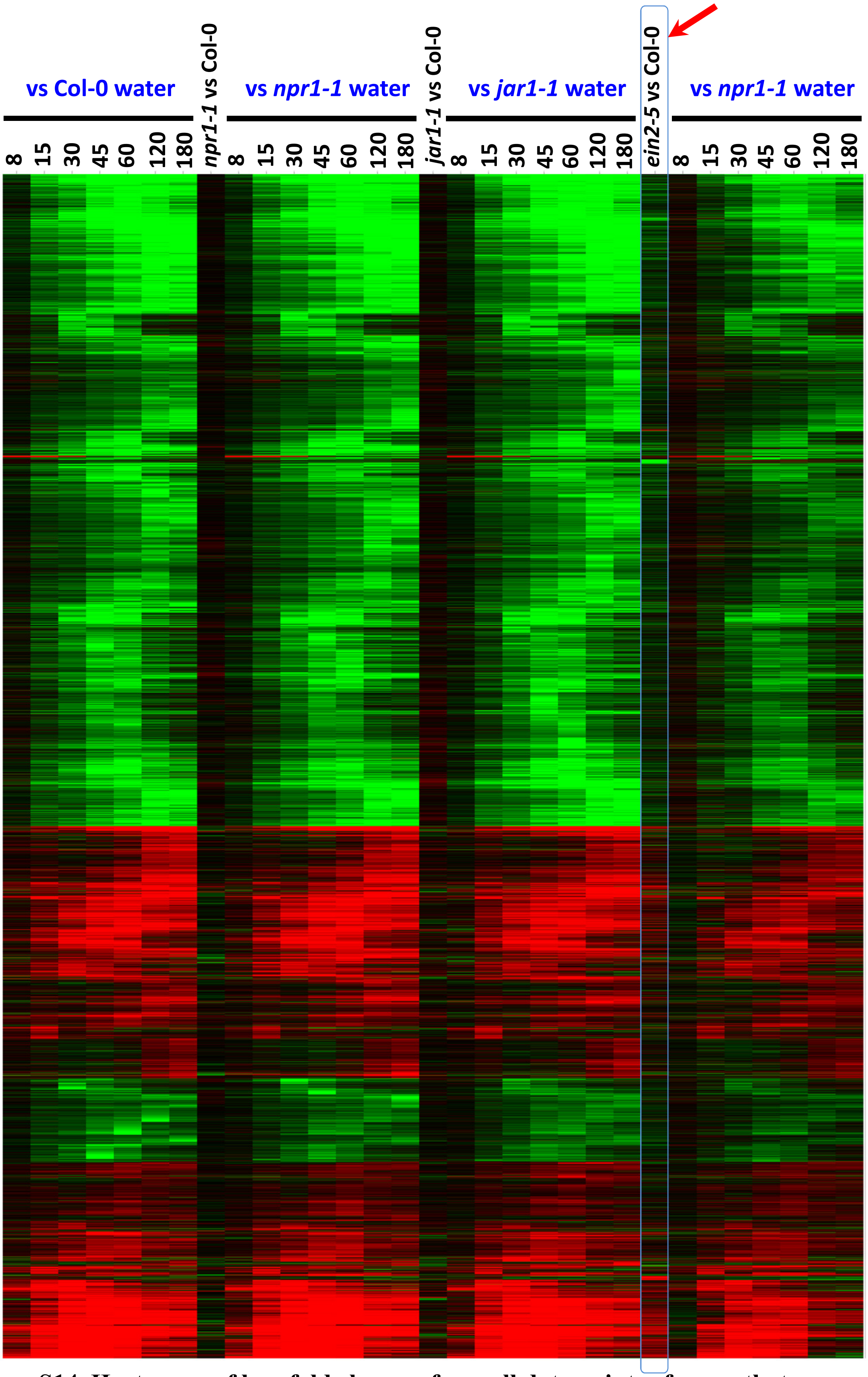

Figure S14. Heat maps of log_2 fold changes from all data points of genes that are differentially expressed at least from one time point of flg22 time course

Log2 fold changes from all data points of 9282 genes presented in Figures S13 are included in the heat map. It is evident that all four genotypes have responded similarly to flg22; while comparison of water treated *ein2-5* with that of Col-0 indicate primed response to flg22 (highlighted in blue block & red arrow). For each genotype flg22 treated samples are compared to water treated sample of respective genotype.

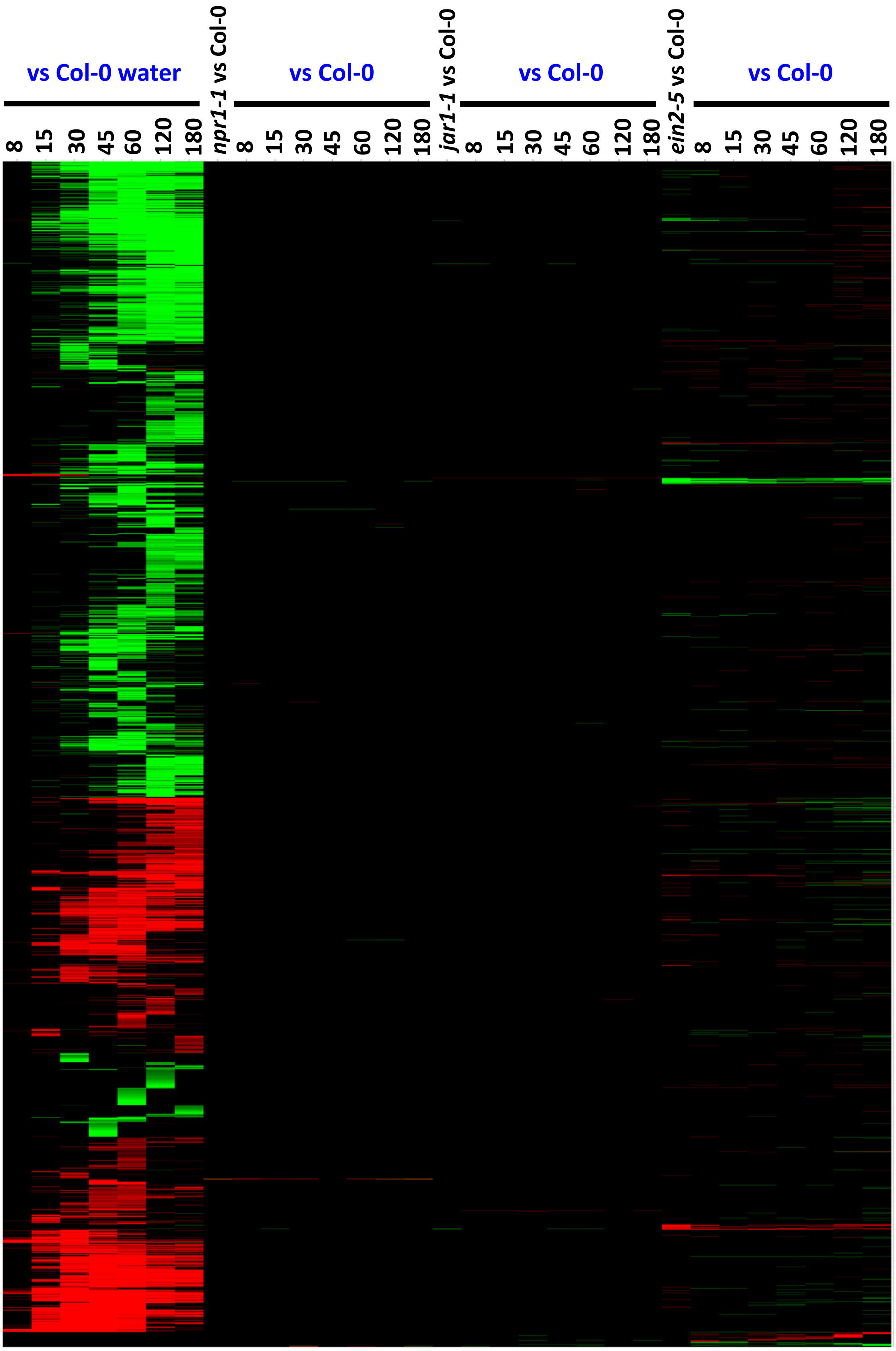

Figure S15. Hierarchical clustering of genes differentially expressed during flg22 time course of four genotypes compared to Col-0.

Heat map showing hierarchically clustered log_2 fold changes of genes differentially expressed during flg22 time course compared to Col-0 (n=7091). Col-0 flg22 time course samples are compared to Col-0 water treated sample (vs Col-0 water), while each mutant genotype (*npr1-1*, *jar1-1* and *ein2-5*) flg22 treated samples are compared to respective flg22 treated time point of Col-0 (vs Col-0). Heat map indicates that flg22 responsive gene expression in the mutants is not significantly different from Col-0, while few genes in ein2-5 show higher induction or repression.

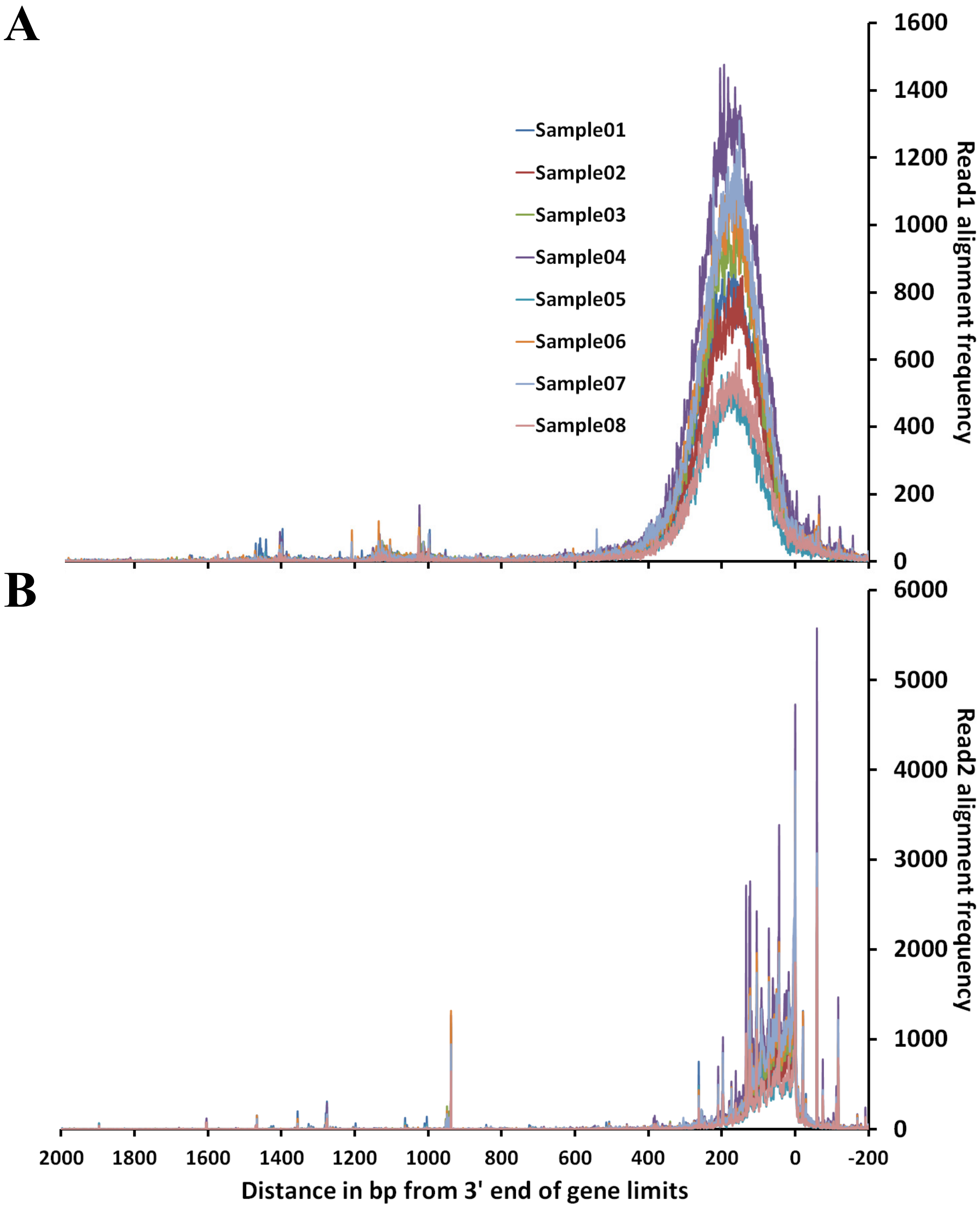

Figure S16. Frequency distribution of Read1 and Read2 from paired end sequencing
Uniquely aligned tags to the sense strand of transcripts on genome sequences from all Arabidopsis genes were used, to plot tag alignment position as a distance from 3’ end of annotated genes against the frequency of reads mapped. (A) Read1 is sequenced within the transcript and a clear peak of read distribution ~200bp from 3’ end is evident, while (B) Read2 is sequenced from the polyadenylation point and major polyadenylation read2 islands (peaks of lines) are observed corresponding to Read1 locations.

A

B

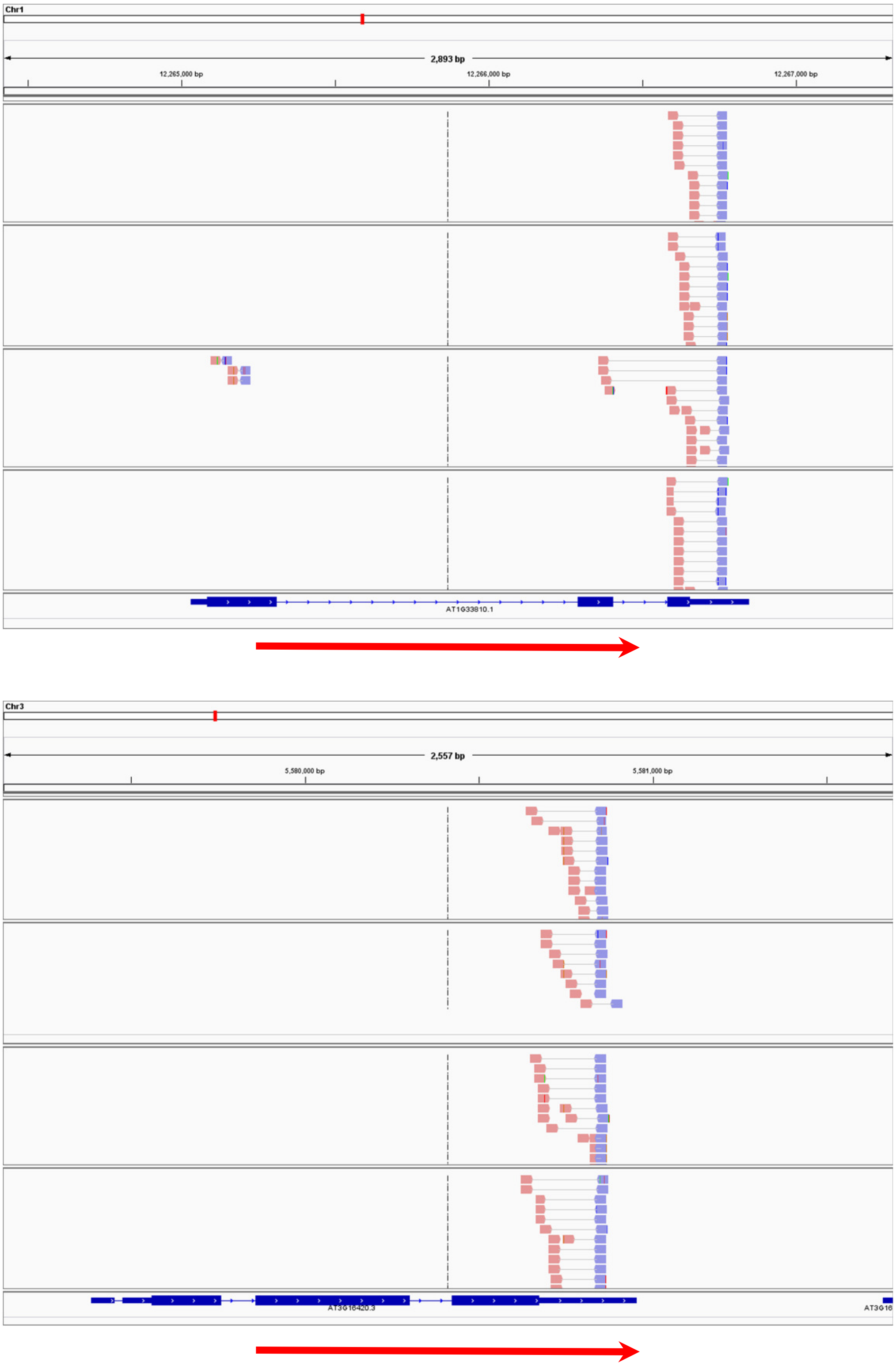

Figure S17. Example showing Read1 and Read2 from paired end sequencing
Paired read alignments are represented on two Arabidopsis gene loci AT1G33810 (A) and AT3G16420 (B). Gene structure is depicted in blue at the bottom of each panel, and gene orientation is represented by a red arrow. Paired reads from 4 independent samples are presented. Read1 is brick red colour and Read2 is light blue colour.

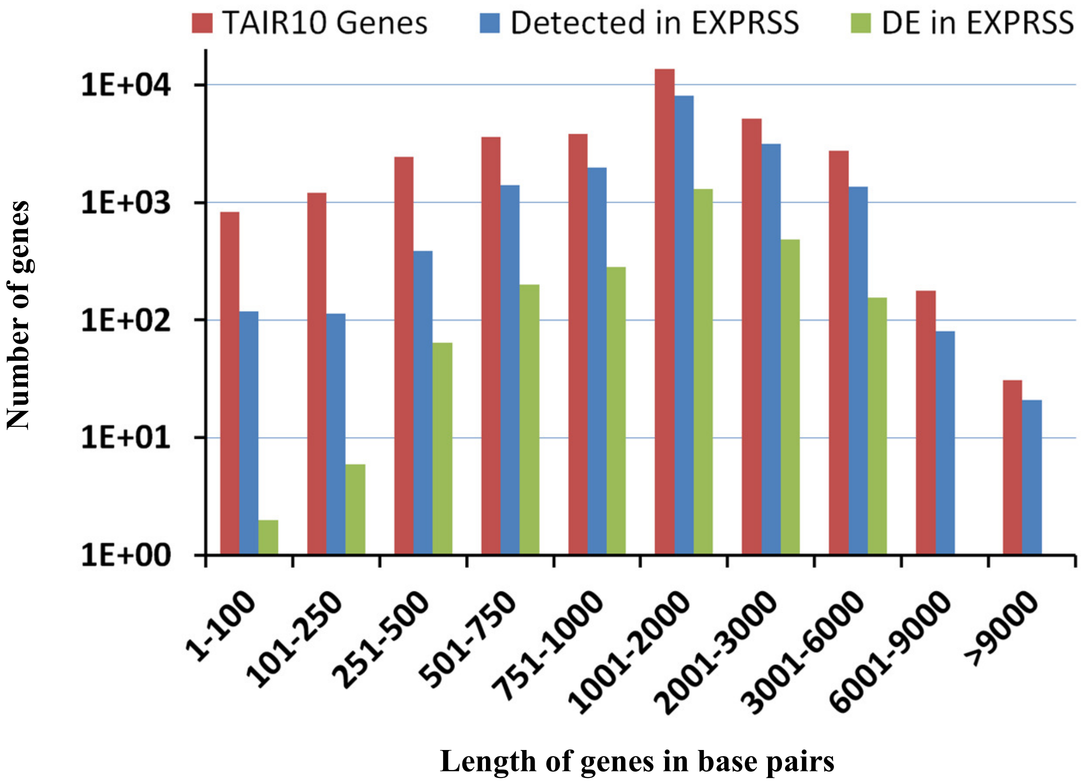

Figure S18. Length distribution of genes detected in EXPRSS
Lengths of the genes present in TAIR10 annotation, detected in EXPRSS and differentially expressed (DE) in EXPRSS are grouped in different length groups and their frequencies are plotted. For genes with multiple variants, the longest transcript is taken into consideration.

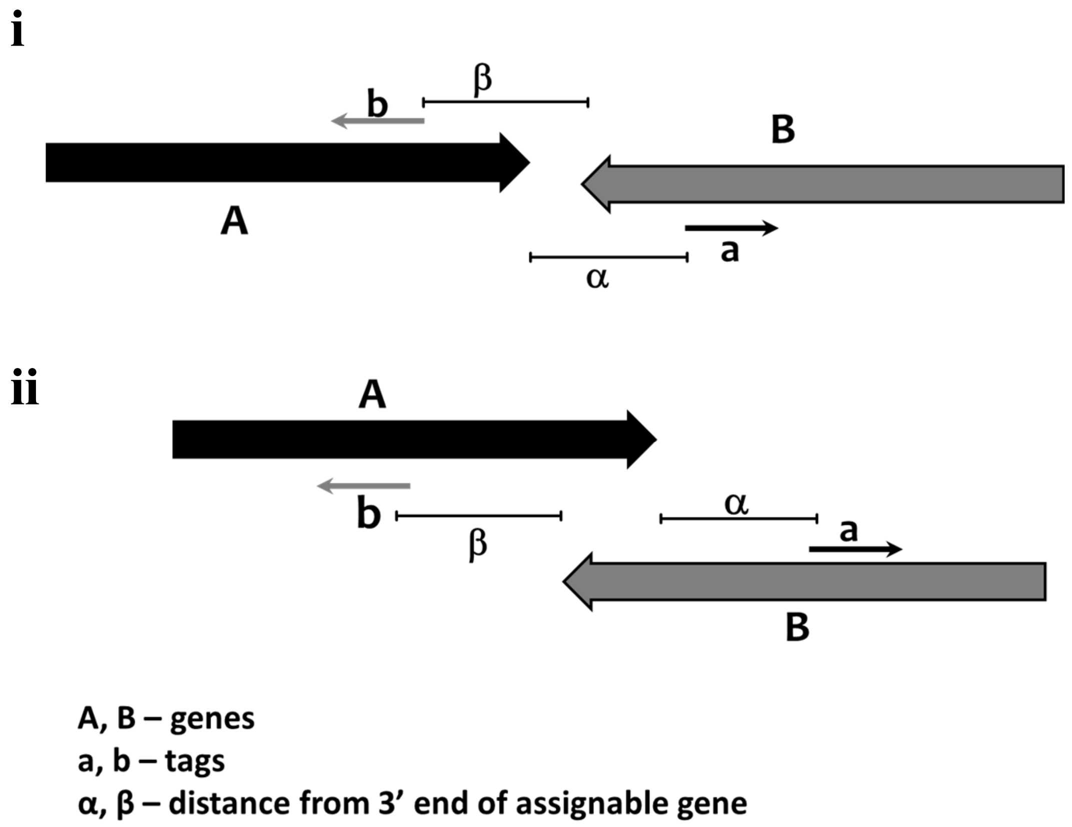

Figure S19. Cartoon depicting tag assignment to genes
Tags aligning as antisense tags to current gene are assigned as sense tags to nearby genes if the distance from 3'end is less than 500bp. (i & ii) Tags a and b are assigned to genes A and B, respectively, if the distances from 3' end of genes to tags (α and β) is less than 500bp.
