# Supplementary material for: EXPRSS: an Illumina based high-throughput expression-profiling method to reveal transcriptional dynamics
Source: BMC Genomics. 2014 May 6;15(1):341. doi: 10.1186/1471-2164-15-341 (PMC4035070; doi:10.1186/1471-2164-15-341)
Supplement: Supplementary file 2 — Additional file 2: Detailed protocols of EXPRSS and NlaIII-DGE and information of primers used. (PDF 142 KB) [file 12864_2013_6041_MOESM2_ESM.pdf]

Supplemental materials and methods

1. EXPRSS Tag-seq single read compatible adapter and primer sequences

(Compatible with single end flow cell only)

Primer Name	Sequence (5' -> 3')	Modification	Purification
oligo-dT_P7	CAAGCAGAAGACGGCATACGACCAGCAGTTTTTT TTTTTTTTTTTTTTTTTTTTTTV*N	*- phosphorothioate bond	HPLC
EXPRSS_Adp Y_top	AATGATACGGCGACCACCGACAGGTTCAGAGTTC TACAGTCCX[3-7]*T	*- phosphorothioate bond	HPLC
EXPRSS_Adp Y_bottom	[Phos]X[3-7] GGACTGTAGAACTCTGAACCACAATAA	5' Phosphorylation	HPLC
EXPRSS_P5-for	AATGATACGGCGACCACCGACA		PAGE
EXPRSS_P7-rev	CAAGCAGAAGACGGCATACGACCA		PAGE
EXPRSS_sequencing_primer	GACCACCGACAGGTTCAGAGTTCTACAGTCC		PAGE
P5-qPCR	AATGATACGGCGACCACCGACA		Desalt
P7-qPCR	CAAGCAGAAGACGGCATACGA		Desalt

X - Indicates the Nucleotides related to barcode

V – A, G & C

N – A, T, G & C

8 barcodes used in this study:

AGC*T, TCG*T, GCTC*T, GTAC*T, TTGGA*T, CGAGA*T, AACAGA*T, CATACG*T

16 barcodes used:

AGC*T, TCG*T, CGG*T, GCA*T, GCTC*T, GTAC*T, ACTG*T, ATCC*T, TTGGA*T, CGAGA*T, GACAC*T, CAGAG*T, CATACG*T, TTGCGC*T, AACAGAG*T, TGTGCGA*T

(*- phosphorothioate bond)

2. EXPRSS Tag-seq paired read compatible adapter and primer sequences

(Compatible with both paired and single end flow cells)

Primer Name	Sequence (5' -> 3')	Modification	Purification
oligo-dT_P7_PE	CAAGCAGAAGACGGCATACGAGATCGGTCTTTTT TTTTTTTTTTTTTTTTTTTTTTTV*N	*- phosphorothioate bond	HPLC
EXPRSS_AdpY_top_PE	AATGATACGGCGACCACCGAGATCTACACTCTTC CCTCACGCACTGATGTTCCX[3-7]*T	*- phosphorothioate bond	HPLC
EXPRSS_AdpY_bottom_PE	[Phos]X[3-7] GGAACATCAGTGCGTGAGGGACATTAC	5' Phosphorylation	HPLC
EXPRSS_P5-PE-for	AATGATACGGCGACCACCGAGA		HPLC
EXPRSS_P7-PE-rev	CAAGCAGAAGACGGCATACGA		HPLC
EXPRSS_sequencing_primer_Read1	GATCTACACTCTTCCCTCACGCACTGATGTTCC		HPLC
EXPRSS_sequencing_primer_Read2	CAAGCAGAAGACGGCATACGAGATCGGTCTTTTT TTTTTTTTTTTTTTTTTTTTTTT		HPLC

X - Indicates the Nucleotides related to barcode

V – A, G & C

N – A, T, G & C

8 barcodes used in this study:

AGC*T, TCG*T, GCTC*T, GTAC*T, TTGGA*T, CGAGA*T, CATACG*T, AACAGAG*T (*- phosphorothioate bond)

3. Buffers

Binding Buffer	20 mM Tris-HCl pH 7.5, 1 M LiCl, 2 mM EDTA
Washing Buffer B	10 mM Tris-HCl pH 7.5, 0.15 M LiCl, 1 mM EDTA
Buffer C	1X PBS, 0.01% Tween20
Cleaning Solution	1X PBS, 1 mM CaCl2, 0.2 mg/ml Proteinase K
Buffer D	1X TE, 0.01% Tween20
EB	10 mM Tris-HCl, pH 8.5
10X annealing buffer	100 mM TRIS pH 8, 10 mM EDTA and 500 mM NaCl

4. Primers used for qPCR verification

Locus	Gene	Orientation	Sequence (5'-> 3')
AT5G60390	EF1A	Forward	CAGGCTGATTGTGCTGTTCTTA
		Reverse	GGTGGTGGCATCCATCTTGTTACA
AT5G64750	ABR1	Forward	GAGGCAACAAAGCCAAACTC
		Reverse	TATGGGCAAAAGGGTAGTCG
AT3G43440	JAZ11	Forward	ACGCATCAACAGTAGCTTGC
		Reverse	AGGCTTCGTGTGGACAAATC
AT3G25882	NIMIN-2	Forward	GGGTTGAGAAACTCGTTGGA
		Reverse	GAAGGGGAAAAACATGAAGGA
AT4G36920	AP2	Forward	AATTCGGCTAATTCGAAGCATA
		Reverse	CGGTTTGACCTAATCCAAGAAC
AT4G39400	BRI1	Forward	CTTGCTTTCCTTCACCACAAC
		Reverse	CCCGGTGTACCAGCTAATGTA
AT2G18170	MPK7	Forward	CTGTAACCGATGCGCTCTTAC
		Reverse	AGATTTCAGCTTCAGGGTGGT
AT3G21630	CERK1	Forward	AGCACACGGTTCCAGTTTATG
		Reverse	TGCTGAACCTCCAACTTCTGT

5. EXPRSS Tag-seq method

First Strand cDNA synthesis: DNaseI treated total RNA (5μg) and 1 μl of 10 μM oligo-dT_P7 primer was diluted with nuclease-free H_20 (20 μl) and incubated at 70°C for 5min. Subsequently 30 μl of First Strand premix consisting of 1X First Strand Buffer, 5 mM DTT, 500 μM dNTP mix, 20U of RNase Inhibitor, 400U of SuperScript II (Concentrations calculated at 50 μl final volume) and nuclease-free H_20 was added and incubated at 50 °C for 1 hour followed by 70 °C for 15 minutes. Tubes were transferred to ice.

Second strand cDNA synthesis: Second strand cDNA synthesis mixture was prepared on ice consisting of 1X *E. coli* ligase buffer, 1X additive (20 mM Tris, 1 mM MgCl2), 1U of RNAseH, 40U of *E. coli* DNA polymerase I, 10U of *E. coli* DNA ligase, 300 μM dNTP (Concentrations calculated at 100 μl final volume) and nuclease free H20 to make up a final volume of 50 μl. This enzyme premix was added to 50 μl of chilled first strand cDNA and incubated at 16°C for 2.5 hours in a Thermomixer. Second strand synthesis was terminated by addition of 6 μl of 0.5 M EDTA (pH 8). RNAse A (1 μl of 10 mg/ml) was added to cDNA reaction and incubated at 37°C for 30 min to degrade RNA in the reaction. Double stranded cDNA prepared was purified by phenol chloroform extraction and precipitated using 1/10th volume of 3M sodium acetate pH 5.2, 20 μg glycogen and two and half volumes of ethanol. Resulting pellet was resuspended in 110 μl of 1X TE.

Covaris shearing of cDNA: Double stranded cDNA (100 μl) was subjected to AFA shearing for 90 seconds with following parameters: Intensity – 5, Duty cycle – 20%. Cycles/Burst – 200. Sheared cDNA was purified using Qiagen PCR purification kit and eluted in 63 μl of EB buffer. Additional volume (10 μl) of purified cDNA can be used to verify candidate gene expression.

End repairing and dA tailing of sheared cDNA: Sheared and purified cDNA was subjected to end repair to convert damaged or incompatible 5'-protruding and/or 3'-protruding ends resulted from shearing to 5'-phosphorylated blunt-end DNA. The reaction mixture for end repairing consists of 60 μl of Eluted DNA, 1X T4 DNA ligase buffer, 0.4 mM dNTP mix, 7U of T4 DNA polymerase, 6U of Klenow DNA polymerase, 20U of T4 Polynucleotide Kinase, 1 mM ATP (Concentrations calculated at 100 μl final volume) and dH20 to make up the final volume. The reaction was carried at 20°C for 30 minutes. End repaired DNA was purified using Qiagen PCR purification kit and was eluted in 35 μl of EB buffer. End repaired DNA was subjected to A-tailing using the reaction consisting of 32 μl of end repaired DNA, 1X NEB2 buffer, 0.2 mM ATP and 15U of Klenow 3' to 5' exo$^-$ (Concentrations calculated at 50 μl final volume) and incubating at 37°C for 40 minutes. DNA was purified by using Qiagen Minelute PCR purification kit and was eluted in 15 μl of EB buffer.

Barcoded Y-shaped Adapter Ligation: End repaired and A-tailed DNA was ligated to Y-shaped Adapter by incubating the reaction mixture consisting of 15 μl of A-tailed DNA, 0.8 μM Y-Adapter, 1X Ligase buffer, 1 mM ATP and 3U of T4 DNA ligase (Concentrations calculated at 30 μl final volume) at 20 °C for 2 hr.

Size selection of library on agarose gel: Ligation was loaded on a 1.5% agarose gel (9 cm) and electrophoresed at 85V for 60 – 80 mins. DNA band of ~300bp was excised using a clean scalpel. Gel extraction was carried using QIAGEN gel extraction kit by following manufacturer's guidelines and eluted in 30 µl EB buffer. Gel slice was incubated in buffer QG at 37 °C instead of 50 °C and left on bench for 5 minutes to re-anneal any denatured fragments.

PCR enrichment of Tag-seq library: PCR enrichment was carried using gel extracted DNA to obtain library sufficient for sequencing. PCR reaction mixture consisted of 1X HF Buffer, 200 µM dNTPs, 200 µM EXPRSS-P5 PCR Primer, 200 µM EXPRSS-P7 PCR Primer, 1U of Phusion DNA polymerase, 4 µl of size selected DNA and H20 to make up the final volume of 50 µl. Amplification was performed using the following PCR programme: Stage 1, 1 minute and 30 seconds at 98 °C, 1 cycle; Stage 2, 20 sec at 98 °C, 20 sec at 65 °C, 30 sec at 72 °C, 15 cycles; Stage 3, 5 min at 72 °C, 1 cycle; hold at 10 °C for 15 minutes. PCR product was purified using QIAGEN PCR purification kit.

6. Adapter preparation

Adapters used for Tag-seq library preparation were made using custom primers in two steps as below.

Step 1: Phosphorylation of Adapter primer: Phosphorylation of adapter primer (EXPRSS_AdpY_bottom) was carried in a 50 µl reaction mixture containing 1X PNK buffer, 10 µM primer, 1 mM ATP, 10U of PNK at 37 ^{o}C for 45 min and at 70 ^{o}C for 15 min.

Step 2: Annealing: Annealing of counterpart primer (EXPRSS_AdpY_top) from the adapter primer pair was carried by providing 1X annealing buffer and 10 µM primer to the above reaction mix and incubating in a thermal cycler using the following step down PCR programme: Stage 1: 5 minutes at 95 ^{o}C, 1 cycle; stage 2: 1 minute 30 seconds at 94 ^{o}C and step down by -1 ^{o}C for each cycle, 65 cycles; stage 3: 4 ^{o}C for 20 minutes.

7. Library quantification adjustment by qPCR

Real-Time qPCR was performed in a 96-well opaque white plate using Chromo4 real-time PCR detection system. All PCR enriched samples were diluted by 1000 times. Standards (previously sequenced library of similar size distribution with known cluster number derived from 10nM library) were diluted to concentrations 100 pM, 10 pM, 1 pM and 0.1 pM and blank. Reactions were carried out for each sample in triplicate and contained 1X SYBR Green JumpStart *Taq* ReadyMix reagent, 2 µl of diluted DNA and 0.625 µM of EXPRSS P5 & P7 primers. The reactions were subjected to PCR with following conditions: Stage 1, 95 °C for 2 min, 1 cycle; stage 2, 95 °C for 20 sec, 65 °C for 20 sec, 72 °C for 30 sec, 35 cycles; stage 3, 72 °C for 5 minutes, 1 cycle. Data analysis was done using Opticon Monitor 3.0 software (Biorad) and amplification plots with a fluorescence threshold above noise were considered to obtain C_T (threshold cycle) values. Concentrations of samples were

estimated using quantity calculations option from software using noise filtered standard values. Mean of three replicates was used as the quantitative estimate of the samples.

8. *Nla*III-DGE tag-seq library preparation (Illumina version)

Libraries were prepared from total RNA using the protocol provided by Illumina [1].

Note: All thermomixer incubations were carried out using following loop program: shaking at 1400 rpm for 15 sec followed by hold for 2 minutes

First Strand cDNA synthesis: Poly(A) RNA was captured from total RNA using Dynabeads oligo-dT(25) magnetic beads. 5 µg of DNaseI treated total RNA was diluted in 50 µl of nuclease free water and heated at 65°C for 5 minutes to disrupt the secondary structures and immediately placed on ice. Dynabeads oligo-dT(25) magnetic beads obtained in PBS, pH 7.4 containing 0.2% NaN3 were re-suspended by gentle flick/vortex and 100 µl were transferred to an nuclease-free siliconized 1.5ml eppendorf tube placed on a magnetic stand. Beads were washed twice with Binding buffer and finally resuspended in 50 µl of Binding buffer. During the entire procedure and subsequently care was taken not to dry the beads. Denatured RNA was added to 50 µl of washed and prepared oligo-dT beads and left to rotate for 10 minutes on a bench top rotary wheel. Upon poly(A) RNA binding beads were washed twice with 200 µl Washing Buffer B and four times with 100 µl 1X First Strand cDNA Synthesis buffer. Washed poly(A) RNA was resuspended well in 50 µl of First Strand premix containing 1X First Strand Buffer, 10 mM DTT, 500 µM dNTP mix, 40U RNase Inhibitor, 400U SuperScript III . Beads were incubated at 50 ^{o}C for 1 hour followed by incubation at 70 ^{o}C for 15 minutes in a Thermomixer and placed on ice for 5-10 minutes.

Second strand cDNA synthesis: Second strand cDNA synthesis mix of 50 µl was prepared on ice containing 1X Second Strand Buffer, 300 µM dNTP mix, 2U RNaseH, 50U DNA Pol I and 10U of *E. coli* DNA ligase (Concentrations calculated at 100 µl final volume). Enzyme premix was added to well chilled first strand cDNA mix and incubated at 16 ^{o}C for 2.5 hours in a Thermomixer. Second strand synthesis was terminated by addition of 6 µl of 0.5 M EDTA and placed on ice. Reaction supernatant was removed by capturing beads on magnetic strand. Beads were washed once with 750 µl of Buffer C then resuspended in 100 µl of Cleaning solution and incubated at 37 ^{o}C for 15 min in a Thermomixer. Beads were washed four times with 750 µl Buffer D followed by two washes with 100 µl 1X NEB Buffer 4. Beads were transferred to a new 1.5 ml siliconized eppendorf tube at final wash before discarding supernatant.

***Nla*III restriction enzyme digestion:** Restriction digestion of the prepared cDNA (from previous step) was done with *Nla*III restriction enzyme in a 100 µl reaction mix containing1X NEB Buffer 4, 0.1 mg BSA, 30U *Nla*IIIin nuclease-free H_20 The reaction mix containing the cDNAbeads was incubated at 37 ^{o}C for 2 hours in a Thermomixer. Next, beads were washed once with 750 µl of Buffer C and resuspended in 100 µl of Cleaning solution and incubated at 37 ^{o}C for 15 min in a Thermomixer. Beads were washed four times with 750 µl Buffer D followed by two

washes with 100 µl 1X T4 DNA ligase buffer. Beads were then transferred to a new 1.5 ml siliconized eppendorf tube and stored at 4 ^{o}C in 1X ligase buffer for approximately 14-16 hr.

Gex Adapter1 ligation: Beads with *Nla*III digested cDNA fragments were briefly resuspended and the 1X ligase buffer was removed by placing the tube on magnetic stand. Adpater1 ligation mix containing 1X Ligation Buffer, 0.6 µM Adapter 1, 5U T4 DNA Ligase and nuclease-free H_20 was added to the beads and incubated at 20 ^{o}C for 2 hours in a Thermomixer. Beads were washed once with 750 µl of Buffer C, resuspended in 100 µl of Cleaning solution and incubated at 37 ^{o}C for 15 min in a Thermomixer. Beads were washed four times with 750 µl Buffer D followed by two washes with 100 µl 1X NEB Buffer 4. After the final wash beads were transferred to a new 1.5 ml siliconized eppendorf tube.

***Mme*I restriction enzyme digestion:** Gex Adapter1 ligated cDNA was digested with MmeI restriction enzyme in a 100 µl reaction mix containing 1X NEB Buffer 4, 50 µM of SAM, 8U of *Mme*I and nuclease-free H_20 with 1.5 hr of incubation at 37°C in a Thermomixer. Supernatant from digestion was transferred to a fresh tube and dephosphorylation reaction was carried out by adding 2 µl of CIAP at 37 °C for 1 hour. Resulting dephosphorylated DNA was phenol/chloroform extracted and ethanol precipitated and was finally resuspended in 10 µl of nuclease-free H_2O.

Gex Adapter2 ligation: MmeI digested and dephosphorylated DNA was ligated to Adapter2 with the reaction mix containing 10 µl of DNA, 150 nM Gex Adapter2, 1X Ligation buffer and 5U T4 DNA ligase and incubated at 20°C for 2 hr.

PCR enrichment of library: PCR enrichment was carried using Gex Adapter2 ligated DNA for obtaining library sufficient for sequencing. PCR reaction mix consisted of 2.5 µl of adapter ligated DNA, 1X HF Buffer,200 µM dNTPs,0.5 µM Gex PCR Primer 1, 0.5 µM Gex PCR Primer 2, 2U Phusion DNA polymerase, and water to makeup a final volume of 50 µl. PCR programme was as follows: stage 1, 30 sec at 98 °C, 1 cycle; stage 2, 10 sec at 98 °C, 30 sec at 60 °C, 15 sec at 72 °C, 20 cycles; stage 2, 10 min at 72 °C, cycle 1; hold at 4 °C.

Size selection and gel extraction of library: Gel electrophoresis of PCR product was carried out on 6% TBE PAGE gels, by loading 50 µl of sample with loading dye and 5 µl of 25 bp DNA ladder, for 35 minutes at 200V. Samples were loaded with a gap of an empty lane between samples. Gel was stained, after lifting from glass gel casette, in TBE/Ethidium Bromide Solution for 10 minutes. DNA band ~85 bp was excised with a clean scalpel. Excised gel piece was placed in a 0.5 ml eppendorf tube, which was punctured with 21 gauge needle at bottom. This tube was placed in an another 1.5 ml eppendorf tube for collecting macerated gel pieces and centrifuged for 2 minutes at 13000 rpm. Two volumes of elution buffer (0.3 M Sodium acetate and 2 mM EDTA pH 8.0) was added to gel pieces and incubated at 37 ^{o}C for 14-16 hrs. Supernatant was collected by centrifuging the gel incubate through Spin-X cellulose filter at 13000rpm for 2minutes DNA was precipitated by addition of 1/10 volume of 3M Sodium acetate (pH 5.2) and 2 volumes of ethanol and DNA was pelleted by

centrifuging 13000 rpm for 20 min and washed once using 70% ethanol. Air dried pellet for 5 minutes and dissolved in 10 µl of EB buffer.

9. *Nla*III-DGE tag-seq library preparation (Modified version)

Libraries were prepared from total RNA by incorporating custom modifications as described below.

Biotinylated and Methylated Adapter1 Ligation: Beads with digested cDNA fragments were briefly resuspended and storage buffer (1X NEB Buffer4) was removed by placing the tube on a magnetic stand. Adpater1 ligation mix consisting of 1X NEB Buffer4, 2 µM of Adapter1, 4U of T4 DNA Ligase, 10U of *Nla*III and nuclease-free H_20 to make final volume of 50 µl was added to the digested cDNA fragments and incubated at 37 ^oC for 2 hours in a Thermomixer. Beads were washed once with 500 µl of Buffer C then resuspended in 200 µl of Cleaning solution and incubated at 37 ^oC for 15 min in a Thermomixer. Beads were washed four times with 200 µl Buffer D followed by two washes with 200 µl 1X NEB Buffer 4. After the final wash beads were transferred to a new 1.5 ml siliconized eppendorf tube.

Preparation of Streptavidin magnetic beads: Streptavidin MagneSphere Paramagnetic Particles (SA-PMPs) obtained in PBS (Promega) were resuspended by gentle flicking and 100 µl (100 µg) of SA-PMPs for each sample were transferred to a siliconized eppendorf tube placed on a magnetic stand. Beads were washed four times with 0.5X SSC and biotinylated DNA (concentration adjusted to 0.5X SSC) was added. This mixture was incubated for 20 min on a bench top rotary wheel and bound fragments were washed using 0.1X SSC for 4 times, by capturing the SA-PMPs on the magnetic stand. Captured fragments after washing, were resuspended in the required buffer as per subsequent use. During the entire procedure and subsequently care was taken not to dry the beads.

Barcoded Adapter2 ligation: Dephosphorylated MmeI digested DNA was captured by SA-PMPs. DNA bound SA-PMPs were washed twice with 100 µl 1X Ligase Buffer and was ligated to Adapter2. The reaction mixture with beads, 2 µM of Barcoded Adapter2, 1X Ligation buffer, 5U of T4 DNA ligase and deionized H20 was incubated at 20°C for 2 hr. Upon completion of ligation beads were washed four times using 0.1X SSC. Beads were then transferred to a new 1.5 ml siliconized eppendorf tube.

PCR enrichment of library: PCR enrichment was carried using Adapter2 ligated DNA beads for obtaining library sufficient for sequencing. Beads were mixed with 50 µl of PCR reaction as carried out for *Nla*III-DGE.

Size selection and gel extraction of library: Gel electrophoresis of PCR product was carried on 8% mini TBE PAGE gel. DNA band (~90 bp) was excised with a clean scalpel and extracted as mentioned for *Nla*III-DGE.

1. Morrissy S, Zhao Y, Delaney A, Asano J, Dhalla N, Li I, McDonald H, Pandoh P, Prabhu A-L, Tam A *et al*: **Digital gene expression by tag sequencing on the illumina genome analyzer.** *Current protocols in human genetics* 2010, **Chapter 11**:Unit 11.11.11-36.
